# Supplementary material for: Are population trends in high-risk alcohol consumption in smokers associated with trends in quit attempts and quit success? A time-series analysis
Source: BMJ Open. 2020 Jul 20;10(7):e034262. doi: 10.1136/bmjopen-2019-034262 (PMC7375511; doi:10.1136/bmjopen-2019-034262)
Supplement: Author's manuscript [file bmjopen-2019-034262.draft_revisions.pdf]

BMJ Open is committed to open peer review. As part of this commitment we make the peer review history of every article we publish publicly available.

When an article is published we post the peer reviewers' comments and the authors' responses online. We also post the versions of the paper that were used during peer review. These are the versions that the peer review comments apply to.

The versions of the paper that follow are the versions that were submitted during the peer review process. They are not the versions of record or the final published versions. They should not be cited or distributed as the published version of this manuscript.

BMJ Open is an open access journal and the full, final, typeset and author-corrected version of record of the manuscript is available on our site with no access controls, subscription charges or pay-per-view fees (http://bmjopen.bmj.com).

If you have any questions on BMJ Open's open peer review process please email info.bmjopen@bmj.com

BMJ Open

Are population trends in high-risk alcohol consumption in smokers associated with trends in quit attempts and quit success? A time series analysis

Journal:	*BMJ Open*
Manuscript ID	bmjopen-2019-034262
Article Type:	Original research
Date Submitted by the Author:	12-Sep-2019

Complete List of Authors:	Beard, Emma; UCL,<br>Brown, Jamie; University College London, Psychology & Language Sciences<br>West, Robert; UCL<br>Michie, Susan; University College London, Centre for Outcomes Research and Effectivenes

Keywords:	time-series, tobacco, alcohol, high-risk drinking, quit attempts

SCHOLARONE™
Manuscripts

I, the Submitting Author has the right to grant and does grant on behalf of all authors of the Work (as defined in the below author licence), an exclusive licence and/or a non-exclusive licence for contributions from authors who are: i) UK Crown employees; ii) where BMJ has agreed a CC-BY licence shall apply, and/or iii) in accordance with the terms applicable for US Federal Government officers or employees acting as part of their official duties; on a worldwide, perpetual, irrevocable, royalty-free basis to BMJ Publishing Group Ltd ("BMJ") its licensees and where the relevant Journal is co-owned by BMJ to the co-owners of the Journal, to publish the Work in this journal and any other BMJ products and to exploit all rights, as set out in our licence.

The Submitting Author accepts and understands that any supply made under these terms is made by BMJ to the Submitting Author unless you are acting as an employee on behalf of your employer or a postgraduate student of an affiliated institution which is paying any applicable article publishing charge ("APC") for Open Access articles. Where the Submitting Author wishes to make the Work available on an Open Access basis (and intends to pay the relevant APC), the terms of reuse of such Open Access shall be governed by a Creative Commons licence – details of these licences and which Creative Commons licence will apply to this Work are set out in our licence referred to above.

Other than as permitted in any relevant BMJ Author's Self Archiving Policies, I confirm this Work has not been accepted for publication elsewhere, is not being considered for publication elsewhere and does not duplicate material already published. I confirm all authors consent to publication of this Work and authorise the granting of this licence.

For peer review only

Are population trends in high-risk alcohol consumption in smokers associated with trends in quit attempts and quit success? A time series analysis

Version 1

Dr Emma Beard[1]
[1] Research Department of Behavioural Science and Health, University College London, London 6BT, UK
e.beard@ucl.ac.uk

Dr Jamie Brown[1]
[1] Research Department of Behavioural Science and Health, University College London, London
jamie.brown@ucl.ac.uk

Professor Robert West[1]
[1] Research Department of Behavioural Science and Health, University College London, London
robert.west@ucl.ac.uk

Professor Susan Michie[2]
[2] Department of Clinical, Educational and Health Psychology, University College London, London
s.michie@ucl.ac.uk

To submit to: BMJ OPEN

Short title: Population trends in high-risk alcohol consumption among smokers

Word count: 2,440

Key words: time-series, tobacco, alcohol, high-risk drinking, quit attempts

Correspondence to: Emma Beard, Research Department of Behavioural Science and Health, University College London, WC1E 6BP. Email: e.beard@ucl.ac.uk. Tel: 02031083179

For peer review only

For peer review only - http://bmjopen.bmj.com/site/about/guidelines.xhtml

Abstract

Objectives: Monthly changes in the prevalence of high-risk drinking and smoking in England appear to be positively correlated. This study aimed to assess how far monthly changes in high-risk drinking were specifically associated with attempts to stop smoking and success of quit attempts.

Design: Data were used from the Alcohol and Smoking Toolkit Studies between April 2014 and June 2018. These involve monthly household face-to-face surveys of representative samples of ~1800 adults.

Setting: England

Participants: Data were aggregated on 88,122 participants over the study period.

Primary and secondary outcome measures: ARIMAX modelling was used to assess the association over time between monthly prevalence of high-risk drinking among smokers and a) prevalence of attempts to quit smoking and b) prevalence of successful quit attempts in those attempting to quit. Bayes Factors (BF) were calculated to compare the null hypothesis with the hypothesis of an effect sufficiently large (β=0.6) to explain the established association between overall prevalence in smoking and high-risk drinking.

Results: No statistically significant associations were found between monthly changes in prevalence of high-risk drinking among smokers and attempts to quit smoking (β=0.156, 95%CI -0.079 to 0.391, p=0.194) or quit success (β=0.066, 95%CI -0.524 to 0.655, p=0.827). Bayes Factors (BF) indicated that the data were insensitive but suggested there is weak evidence for the null hypothesis in the case of both quit attempts (BF=0.80) and quit success (BF=0.53).

Conclusions: Monthly changes in prevalence of high-risk alcohol consumption in England are not clearly associated with changes in quit attempt or quit success rates.

Strengths and limitations of this study

- This is the first time series study to assess how far monthly changes in high-risk drinking are associated with attempts to stop smoking and success of quit attempts.

For peer review only - http://bmjopen.bmj.com/site/about/guidelines.xhtml

- This study uses a large representative sample of the population in England.
- In countries with weaker tobacco control different effects may be observed.
- Data are observational and so strong conclusions regarding cause and effect cannot be made.

For peer review only

Background

In England, around 15% of the population are smokers and 20% drink alcohol at high-risk levels, i.e. levels which are likely to cause harm (1, 2). Both are associated with a number of preventable conditions and appear to have an accumulative effect on the risk of mortality(3). The association between high-risk drinking and smoking has been well established at an individual level. High-risk drinkers are substantially more likely to smoke (4-8) and smokers who report starting a quit attempt also report lower alcohol consumption (9, 10). Attempts to quit smoking are also less successful among those with an alcohol use disorder(11-13). Such associations may arise by a number of mechanisms. For example, smokers drinking at high-risk levels may follow advice that it is important to restrict alcohol consumption when they quit(9, 14-16), alcohol and smoking appear to provide cues to lapses for the other and there may be pharmacological interactions between nicotine and alcohol (17-19). This is contrary to the popular notion of self-medication and reward seeking with people deprived of cigarettes compensating by increasing their use of alcohol (20).

It is important to identify whether similar patterns exist at a population level. An association in either direction could mean that policies that reduce smoking prevalence may have the added benefit of reducing high-risk drinking or vice versa. In England, since 2014 monthly data have been gathered on high-risk drinking, smoking status, attempts to quit smoking and quit success (21). Recently, we used these data to examine population-level associations over time between smoking and high-risk drinking and showed that monthly changes in prevalence of smoking in England were associated positively with prevalence of high-risk drinking. However, there were no significant associations between motivation to stop and motivation to reduce alcohol consumption, or attempts to quit smoking and attempts to reduce alcohol consumption(22). We found the combination of results surprising and suggested that the association with overall prevalence may be related to an unmeasured variable that accounted for the change in both smoking and high-risk drinking. Alternatively, the failure to find an overall association between motivation and attempts for each

For peer review only - http://bmjopen.bmj.com/site/about/guidelines.xhtml

behaviour may be an issue of power when focussing on the global association between subsamples that represented only a fifth of the overall sample.

This study attempted to resolve this apparent contradiction and extend these findings by relying on the assessment of specific trends expected to be more strongly related, if the association between the prevalence of smoking and high-drinking is causal. Specifically, we will assess whether changes in trends of excessive alcohol consumption *among smokers* are associated with trends in attempts to quit smoking and quit success. If no association is found, this would support the conclusion of a third unmeasured variable associated with both smoking and high-risk drinking.

This study addressed the following research questions:

1. Is there an association in England between increases or decreases in monthly prevalence of high-risk drinking among smokers and attempts to quit smoking?
2. Is there an association in England between increases or decreases in monthly prevalence of high-risk drinking among smokers and quit success rates?

Methods

Study design

Data were used from the Smoking and Alcohol Toolkit Studies (STS and ATS) collected between April 2014 and June 2018. The STS and ATS are ongoing studies that involve a series of monthly cross-sectional household, face-to-face, computer assisted surveys of representative samples of ~1800 adults in England aged 16+. Thus, the same participants take part in both surveys. The respondents are recruited using a type of random location sampling, which is a hybrid between random probability and simple quota sampling. England is first split into over 170000 'Output Areas', comprising of approximately 300 households. These areas are then stratified according to

For peer review only - http://bmjopen.bmj.com/site/about/guidelines.xhtml

ACORN characteristics and geographic region (http://www.caci.co.uk/acorn/) and are randomly allocated to interviewers. Interviewers visit households within their allocated locality starting at a random point in the area. One member per household is interviewed until interviewers achieve local quotas designed to minimise differences in the probability of participation. Participants appear to be representative of the population in England, having similar socio-demographic composition and smoking characteristics to large national surveys based on probability samples such as the Health Survey for England (23), while drinking characteristics also appear similar at a regional level to other national surveys (24). For further details see: www.smokinginengland.info and www.alcoholinengland.info and the published protocol (21, 23).

Participants

Data were collected on 88,122 participants over the study period. Of these, 19.94% (95%CI 19.67 to 20.20 n=17,560) reported that they had smoked in the past year. Data from these participants were aggregated monthly and this forms the basis of the sample in this paper.

Measures

Participants were asked whether they smoked or had smoked cigarettes (including hand-rolled) daily or non-daily in the past year and to complete the Alcohol Use Disorders Identification Test (AUDIT) (25). The AUDIT identifies people who could be classed as dependent, harmful or hazardous drinkers and has demonstrated validity, high internal consistency and good test-retest reliability across gender, age and cultures (26-29). Those scoring 8 or more were classed as high-risk drinkers. This is a common threshold for high-risk drinkers (27, 30-32). The prevalence of high-risk alcohol consumption among smokers in each month was obtained by counting the number smokers reporting an AUDIT score greater than or equal to 8.

Past year smokers were then asked:

For peer review only - http://bmjopen.bmj.com/site/about/guidelines.xhtml

1. “How many serious attempts to stop smoking have you made in the last 12 months? By serious attempt I mean you decided that you would try to make sure you never smoked again. Please include any attempt that you are currently making and please include any successful attempt made within the last year”.
2. “How long did your most recent serious quit attempt last before you went back to smoking?”

The prevalence of quit attempts in monthly were calculated as the number of respondents who reported having made one or more quit attempts in the past 12 months divided by the number of past year smokers. The success rate in each quarter was calculated as the number of respondents reporting that they were still not smoking divided by the number reporting having made a quit attempt.

Analysis

The analysis plan, data and syntax were preregistered on the Open Science Framework (https://osf.io/384gx/). Cases with missing data on either smoking or drinking variables were classified as missing in calculating the prevalence figures: smoking status (n=55; %=0.06), high-risk drinking status among smokers (n=202; %=1.17) and quit attempts among smokers (n=562; %=3.24). All data were analysed in R studio.

Data were weighted (see (23) for further details) to match the population in England and analysed using Autoregressive Integrated Moving Average with Exogeneous Input (ARIMAX) modelling to assess the association between prevalence of high risk drinking among smokers and 1) prevalence of attempts to quit smoking and 2) prevalence of successful attempts to quit smoking among those having made a quit attempt. ARIMAX is an extension of autoregressive integrated moving average analysis (ARIMA), which produces forecasts based upon prior values in the time series

For peer review only - http://bmjopen.bmj.com/site/about/guidelines.xhtml

(Autoregressive terms; AR) and the errors made by previous predictions (Moving Average terms; MA). We followed a standard ARIMAX modelling approach (33).

The ARIMAX assumption of weak exogeneity was met: past prevalence of quit attempts (p=0.747) and quit success (p=0.999) did not statistically predict future prevalence of high-risk drinking among smokers. No outliers were identified in any of the series using an approach based on that described by Chen and Liu(34, 35). To stabilise the variance the series were log-transformed. The Augmented Dickey-Fuller test and visual inspection of the plots indicated that first order differencing was required for both time series. First order differencing involves calculating the change between one observation and the next. No additional seasonal differencing was required (36).

The autocorrelation and partial autocorrelation functions were examined to determine the non-seasonal MA and AR terms. These suggested an ARIMAX(0,1,1) model for the time series predicting both prevalence of quit attempts and prevalence of quit success. This was confirmed by comparing models with different specifications using the AIC. To identify the most appropriate transfer function for the continuous explanatory variables the sample cross-correlation function was checked and models with varying distributed lags compared using the Akaike Information Criterion. This suggested a lag of 0 when predicting the prevalence of quit attempts and predicting the prevalence of quit success, thus only current values and not lagged (past period) values of the input series were used to predict current values of the output series. In our previous study, prevalence of smoking was found to be associated with high-risk drinking with a distributed lag of 2 (22). Thus, additional sensitivity analyses were run with the output series lagged by an order of 2 i.e. the time base was shifted back by 2 months.

For peer review only - http://bmjopen.bmj.com/site/about/guidelines.xhtml

The Ljung-Box test for white noise showed that the residuals for both fitted models were free of serial correlation. A number of additional model checks were also made. First, the autocorrelation terms included in the model were checked for their statistical significance. Secondly, it was determined whether the model residuals were normally distributed, random and independent. Finally, that the inclusion of the MA term conformed to the bounds of invertibility i.e. its value was <1 (33, 34).

Bayes factors (BFs) were derived for non-significant findings using an online calculator to disentangle whether there is evidence for the null hypothesis of no effect (BF <1/3rd) or the data are insensitive (BF between 1/3rd and 3)(37, 38). A half-normal distribution was assumed with a percentage change in the outcomes of interest for every percentage increase in the input series of 0.6%. This is on the basis of a previous study showing that smokers who had made a quit attempt were around 40% less likely to report that they were high risk drinkers (9). Strengthening the Reporting of Observational Studies in Epidemiology (STROBE) guidelines for the reporting of observational studies were followed throughout (39).

Patient involvement

No patients were involved in setting the research question or the outcome measures, nor were they involved in developing plans for recruitment, design, or implementation of the study. No patients were asked to advise on interpretation or writing up of results. There are no plans to disseminate the results of the research directly to study participants or any specific patient community.

Results

Figure 1 shows the raw time series data from 2014 to 2018. Prevalence of high-risk drinking among smokers declined from 26.9% (95% CI 22.34 to 32.03) in 2014 to 23.7% (95% CI 19.26 to 28.87) in June 2018. Attempts to quit smoking also declined from 38.1% (95%CI 32.86 to 43.66) to 28.5%

For peer review only - http://bmjopen.bmj.com/site/about/guidelines.xhtml

(95%CI 23.60 to 33.90) and quit success from 19.6% (95%CI 13.22 to 27.87) to 9.4% (95%CI 4.51 to 17.95) in June 2018.

Tables 1 shows the results of the ARIMAX models assessing the association between prevalence of high-risk drinking among smokers and (1) quit attempts and (2) quit success. The findings were inconclusive as to whether any associations were present. BFs suggested that there is anecdotal evidence for the null hypothesis that prevalence of high-risk drinking among smokers is not associated with prevalence of quit attempts and prevalence of quit success. Findings were similar when a 2 month back shifted lag was used for prevalence of quit attempts and quit success.

Discussion

To our knowledge, this is the first empirical study to estimate the population association between high-risk drinking among smokers and attempts to quit smoking and the success of those attempts. There was weak evidence that there was no substantial association between changes in the prevalence of high-risk drinking and quit attempts and quit success.

These findings appear to be at odds with individual level studies which suggest that smokers with an alcohol use disorder are less likely to attempt and succeed in stopping smoking (12, 13). Alcohol consumption during attempts at smoking cessation is also associated with a greater risk of relapse (14). As a result, smokers are often advised to lower their alcohol consumption when they attempt to quit smoking(9). Of course, it remains plausible that high-risk drinking among smokers may still be associated with a small effect on mean population prevalence of quit attempts and their success, but it was not possible to detect this in the current study. An association may also be masked by factors impacting at a population level which were not accounted for in the current study. Although we are unaware of any major population-level interventions or other events during the study period which may have affected the associations under investigation, we cannot rule out residual confounding. There may also be some statistical bias due to the loss of power and sensitivity that

For peer review only - http://bmjopen.bmj.com/site/about/guidelines.xhtml

comes with aggregating data. Prevalence of high-risk drinking among smokers will also be somewhat noisier than if prevalence was also assessed among non-smokers, given the smaller sample size involved in estimation.

These findings suggest that the previously identified positive association between prevalence of smoking and prevalence of high-risk drinking is unlikely to be causal, whereby smokers attempting to quit, and those succeeding, also reduce their alcohol intake (22). Although it remains possible that use of alcohol by smokers impacts on other key indices including longer term abstinence, the small proportion of smokers who relapse long term (i.e. after a year) could not account for the size of association noted. It may instead be that overall prevalence is related to an unmeasured variable, perhaps economic factors and sociocultural events, that account for the change in both smoking and high-risk drinking. For example, in recent years taxation on cigarettes and alcohol have increased linearly, driving down sales of both (40, 41). There have also been substantial fluctuations in average household income since 2013, which have been shown to independently affect smoking and alcohol consumption (42-44). Sporting events such as the Olympics may also concurrently increase alcohol and tobacco intake as they are celebratory occasions.

A strength of this study is the use of a large representative sample of the population in England. Several limitations need to be considered. First, the ATS required participants to recall their alcohol consumption and attempts to quit smoking which is likely to have been somewhat inaccurate due to recall bias and social desirability. For example, it has been found that a large proportion of unsuccessful quit attempts fail to be reported, particularly if they only last a short time or occurred longer ago(45). However, social pressure in population surveys tends to be low and so it is generally considered acceptable to rely on self-report data(46). Second, these findings may not generalise to other countries. England has a strong tobacco-control climate. In countries with weaker tobacco control or different alcohol control policies, different effects may be observed.

For peer review only - http://bmjopen.bmj.com/site/about/guidelines.xhtml

Conclusion

These findings suggest that the previously identified positive association between prevalence of smoking and prevalence of high-risk drinking is unlikely to be causal, whereby smokers attempting to quit, and those succeeding, also reduce their alcohol intake. Instead, it may be that overall prevalence is related to an unmeasured third variable such as economic factors and sociocultural events.

For peer review only

For peer review only - http://bmjopen.bmj.com/site/about/guidelines.xhtml

List of abbreviations
ARIMAX - autoregressive integrated moving average with exogenous input
BF – Bayes Factor
ATS – Alcohol Toolkit Study
STS – Smoking Toolkit Study

For peer review only

Table 1: Estimated percentage point changes in proportion of quit attempts and proportion of quitters who met criteria for quit success during the study period, based on autoregressive integrated moving average with exogenous input (ARIMAX) models

		Output series					
		Quit attempts[1]			Quit success[2]		
		Percentage change per 1% change in the exposure	95%CI	p	Percentage change per 1% change in the exposure	95%CI	p
Input series	**Model 1:** High-risk drinking among smokers (no backward lag of the output series)	0.156	-0.079 to 0.391	0.194	0.066	-0.524 to 0.655	0.827
	**Model 2:** High-risk drinking among smokers (two month backward lag of the output series)	0.065	-0.183 to 0.313	0.608	0.134	-0.469 to 0.736	0.663
	**Bayes Factor**						
	Model 1	0.80			0.53		
	Model 2	0.33			0.64		

Declarations

Ethical approval and consent to participate

Ethics approval for the Smoking Toolkit Survey (STS) was originally granted by the UCL Ethics Committee (ID 0498/001) and approval for the ATS was granted by the same committee as an extension of the STS (ID 2808/005). In accordance with our ethical approval, all respondents were given a written information sheet about the study, and provided informed verbal consent

Availability of data and material

The analysis plan, data and syntax were preregistered on the Open Science Framework (https://osf.io/384gx/).

Funding

The Smoking Toolkit Study is currently primarily funded by Cancer Research UK (C1417/A14135; C36048/A11654; C44576/A19501) and has previously also been funded by Pfizer, GSK, and the Department of Health. The ATS is currently funded by the NIHR School for Public Health Research (SPHR) (SPHR-SWP-ALC-WP5). SPHR is a partnership between the Universities of Sheffield; Bristol; Cambridge; Exeter; UCL; The London School for Hygiene and Tropical Medicine; the LiLaC collaboration between the Universities of Liverpool and Lancaster and Fuse; The Centre for Translational Research in Public Health, a collaboration between Newcastle, Durham, Northumbria, Sunderland and Teesside Universities. The views expressed are those of the authors(s) and not necessarily those of the NHS, NIHR, or Department of Health. No funders had any involvement in the design of the study, the analysis or interpretation of the data, the writing of the report, or the decision to submit the paper for publication. JB's post is funded CRUK (C1417/A14135). RW is funded by Cancer Research UK (C1417/A14135). EB is funded by the NIHR SPHR (SPHR-SWP-ALC-WP5) and CRUK also provide support (C1417/A14135).

For peer review only - http://bmjopen.bmj.com/site/about/guidelines.xhtml

Competing interests

RW undertakes consultancy and research for and receives travel funds and hospitality from manufacturers of smoking cessation medications. RW salary is funded by Cancer Research UK. SM receives support from Cancer Research UK and the National Institute for Health Research (NIHR)'s School for Public Health Research (SPHR). EB and JB have received unrestricted research funding from Pfizer. PM's research is funded by a variety of governmental funding agencies including UKRI and NIHR.

Authors' contributions

EB, JB, SM and RW wrote the first draft of the manuscript and conducted the analysis. All other authors commented on this draft and contributed to the final version. All authors read and approved the final manuscript.

For peer review only

References

1. Statistics OfN. Adult smoking habits in the UK: 2017. 2018.
2. Health and Social Care Information Centre. Adult Psychiatric Morbidity Survey 2014: Alcohol Dependence (Chapter 10) 2016. Available from: http://content.digital.nhs.uk/catalogue/PUB21748/apms-2014-alcohol.pdf.
3. Kvaavik E, Batty GD, Ursin G, Huxley R, Gale CR. Influence of individual and combined health behaviors on total and cause-specific mortality in men and women: the United Kingdom health and lifestyle survey. Archives of internal medicine. 2010;170(8):711-8.
4. Lasser K, Boyd JW, Woolhandler S, Himmelstein DU, McCormick D, Bor DH. Smoking and mental illness: a population-based prevalence study. JAMA. 2000;284.
5. Falk DE, Yi HY, Hiller-Sturmhofel S. An epidemiologic analysis of co-occurring alcohol and tobacco use and disorders: findings from the National Epidemiologic Survey on Alcohol and Related Conditions. Alcohol Res Health. 2006;29.
6. Kalman D, Morissette SB, George TP. Co-morbidity of smoking in patients with psychiatric and substance use disorders. Am J Addict. 2005;14.
7. Kessler RC, Chiu WT, Demler O, Merikangas KR, Walters EE. Prevalence, severity, and comorbidity of 12-month DSM-IV disorders in the National Comorbidity Survey Replication. Arch Gen Psychiatry. 2005;62.
8. Hasin DS, Stinson FS, Ogburn E, Grant BF. Prevalence, correlates, disability, and comorbidity of DSM-IV alcohol abuse and dependence in the United States: results from the National Epidemiologic Survey on Alcohol and Related Conditions. Arch Gen Psychiatry. 2007;64.
9. Brown J, West R, Beard E, Brennan A, Drummond C, Gillespie D, et al. Are recent attempts to quit smoking associated with reduced drinking in England? A cross-sectional population survey. BMC Public Health. 2016;16(1):1-7.
10. Przulj D, Hajek P, Snuggs S, McRobbie H. Changes in Alcohol Consumption During a Stop-Smoking Attempt and Differences Between Smokers Using Nicotine Replacement and Smokers Using Varenicline. Nicotine Tob Res. 2018;20(5):583-8.
11. Hughes JR, Kalman D. Do smokers with alcohol problems have more difficulty quitting? Drug Alcohol Depend. 2006;82.
12. Weinberger AH, Pilver CE, Hoff RA, Mazure CM, McKee SA. Changes in smoking for adults with and without alcohol and drug use disorders: longitudinal evaluation in the U.S. population. Am J Drug Alcohol Abuse. 2013;39.
13. Leeman RF, McKee SA, Toll BA, Krishnan-Sarin S, Cooney JL, Makuch RW, et al. Risk factors for treatment failure in smokers: relationship to alcohol use and to lifetime history of an alcohol use disorder. Nicotine Tob Res. 2008;10.
14. Kahler CW, Spillane NS, Metrik J. Alcohol use and initial smoking lapses among heavy drinkers in smoking cessation treatment. Nicotine Tob Res. 2010;12.
15. Gulliver SB, Kamholz BW, Helstrom AW. Smoking cessation and alcohol abstinence: what do the data tell us? Alcohol Res Health. 2006;29.
16. Friend KB, Pagano ME. Smoking cessation and alcohol consumption in individuals in treatment for alcohol use disorders. J Addict Dis. 2005;24.
17. Rose JE, Brauer LH, Behm FM, Cramblett M, Calkins K, Lawhon D. Psychopharmacological interactions between nicotine and ethanol. Nicotine & Tobacco Research. 2004;6(1):133-44.
18. Field M, Mogg K, Bradley BP. Alcohol increases cognitive biases for smoking cues in smokers. Psychopharmacology. 2005;180(1):63-72.
19. Verplaetse TL, McKee SA. An overview of alcohol and tobacco/nicotine interactions in the human laboratory. The American journal of drug and alcohol abuse. 2017;43(2):186-96.
20. Blume AW, Schmaling KB, Marlatt GA. Revisiting the self-medication hypothesis from a behavioral perspective. Cognitive and Behavioral Practice. 2000;7(4):379-84.

For peer review only - http://bmjopen.bmj.com/site/about/guidelines.xhtml

21. Beard E, Brown J, West R, Acton C, Brennan A, Drummond C, et al. Protocol for a national monthly survey of alcohol use in England with 6-month follow-up:'The Alcohol Toolkit Study'. BMC public health. 2015;15(1):230.
22. Beard E, West R, Michie S, Brown J. Association between smoking and alcohol-related behaviours: a time–series analysis of population trends in England. Addiction (Abingdon, England). 2017;112(10):1832-41.
23. Fidler JA, Shahab L, West O, Jarvis MJ, McEwen A, Stapleton JA, et al. 'The smoking toolkit study': a national study of smoking and smoking cessation in England. BMC Public Health. 2011;11(1):479.
24. Beard E, Brown J, West R, Angus C, Kaner E, Michie S. Healthier central England or North–South divide? Analysis of national survey data on smoking and high-risk drinking. BMJ Open. 2017;7(3).
25. Casswell S, Meier P, MacKintosh AM, Brown A, Hastings G, Thamarangsi T, et al. The International Alcohol Control (IAC) Study—Evaluating the Impact of Alcohol Policies. Alcoholism: Clinical and Experimental Research. 2012;36(8):1462-7.
26. Saunders JB, Aasland OG, Amundsen A, Grant M. Alcohol consumption and related problems among primary health care patients: WHO collaborative project on early detection of persons with harmful alcohol consumption--I. Addiction (Abingdon, England). 1993;88(3):349-62.
27. Saunders JB, Aasland OG, Babor TF, de la Fuente JR, Grant M. Development of the Alcohol Use Disorders Identification Test (AUDIT): WHO Collaborative Project on Early Detection of Persons with Harmful Alcohol Consumption--II. Addiction (Abingdon, England). 1993;88(6):791-804.
28. Allen JP, Litten RZ, Fertig JB, Babor T. A Review of Research on the Alcohol Use Disorders Identification Test (AUDIT). Alcoholism: Clinical and Experimental Research. 1997;21(4):613-9.
29. Hays R, Merz J, Nicholas R. Response burden, reliability, and validity of the CAGE, Short MAST, and AUDIT alcohol screening measures. Behavior Research Methods, Instruments, & Computers. 1995;27(2):277-80.
30. Caviness CM, Hatgis C, Anderson BJ, Rosengard C, Kiene SM, Friedmann PD, et al. Three Brief Alcohol Screens for Detecting Hazardous Drinking in Incarcerated Women. Journal of Studies on Alcohol and Drugs. 2009;70(1):50-4.
31. National Institute on Alcohol Abuse and Alcoholism. Helping Patients Who Drink Too Much: A Clinician's Guide. Bethesda, MD: National Institute on Alcohol Abuse and Alcoholism, 2007.
32. Kokotailo PK, Egan J, Gangnon R, Brown D, Mundt M, Fleming M. Validity of the alcohol use disorders identification test in college students. Alcoholism: Clinical and Experimental Research. 2004;28(6):914-20.
33. Box GE, Jenkins GM, Reinsel GC. Time series analysis: forecasting and control: John Wiley & Sons; 2011.
34. Chen C, Liu L-M. Joint estimation of model parameters and outlier effects in time series. Journal of the American Statistical Association. 1993;88(421):284-97.
35. López-de-Lacalle J. tsoutliers R Package for Automatic Detection of Outliers in Time Series.
36. Dickey DA, Fuller WA. Distribution of the estimators for autoregressive time series with a unit root. Journal of the American statistical association. 1979;74(366a):427-31.
37. Dienes Z. Using Bayes to get the most out of non-significant results. Frontiers in Psychology. 2014;5(781).
38. Beard E, Dienes Z, Muirhead C, West R. Using Bayes factors for testing hypotheses about intervention effectiveness in addictions research. Addiction (Abingdon, England). 2016;111(12):2230-47.

For peer review only - http://bmjopen.bmj.com/site/about/guidelines.xhtml

39. Von Elm E, Altman DG, Egger M, Pocock SJ, Gøtzsche PC, Vandenbroucke JP, et al. The Strengthening the Reporting of Observational Studies in Epidemiology (STROBE) statement: guidelines for reporting observational studies. PLoS medicine. 2007;4(10):e296.
40. Institute of Alcohol Studies. How have UK alcohol taxes changed over time? 2017. Available from: http://www.ias.org.uk/Alcohol-knowledge-centre/Price/Factsheets/How-have-UK-alcohol-taxes-changed-over-time.aspx.
41. ASH. Timeline of changes in tobacco taxation in the UK from 1993 to the present 2017. Available from: http://ash.org.uk/information-and-resources/taxation-illicit-trade/taxation/ash-analysis-of-tobacco-tax-increases-in-the-united-kingdom/.
42. Cerdá M, Johnson-Lawrence V, Galea S. Lifetime income patterns and alcohol consumption: Investigating the association between long- and short-term income trajectories and drinking. Social science & medicine (1982). 2011;73(8):1178-85.
43. Perelman J, Alves J, Pfoertner TK, Moor I, Federico B, Kuipers MAG, et al. The association between personal income and smoking among adolescents: a study in six European cities. Addiction (Abingdon, England). 2017;112(12):2248-56.
44. ONS. Nowcasting household income in the UK: financial year ending 2017. 2018.
45. Berg CJ, An LC, Kirch M, Guo H, Thomas JL, Patten CA, et al. Failure to report attempts to quit smoking. Addictive behaviors. 2010;35(10):900-4.
46. Wong SL, Shields M, Leatherdale S, Malaison E, Hammond D. Assessment of validity of self-reported smoking status. Health reports. 2012;23(1):D1.

Figure 1: Time series showing the prevalence of a) high risk drinking; b) attempts to quit smoking among smokers and c) successful quit attempts among smokers having made a quit attempt

For peer review only

For peer review only - http://bmjopen.bmj.com/site/about/guidelines.xhtml

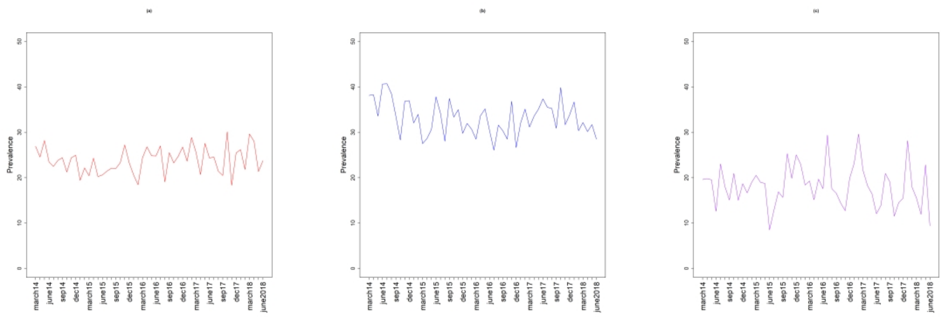

762x254mm (300 x 300 DPI)

STROBE 2007 (v4) Statement—Checklist of items that should be included in reports of *cross-sectional studies*

Section/Topic	Item #	Recommendation	Reported on page #
**Title and abstract**	1	(*a*) Indicate the study's design with a commonly used term in the title or the abstract	1
		(*b*) Provide in the abstract an informative and balanced summary of what was done and what was found	2
**Introduction**			
Background/rationale	2	Explain the scientific background and rationale for the investigation being reported	4-5
Objectives	3	State specific objectives, including any prespecified hypotheses	5
**Methods**			
Study design	4	Present key elements of study design early in the paper	6
Setting	5	Describe the setting, locations, and relevant dates, including periods of recruitment, exposure, follow-up, and data collection	6
Participants	6	(*a*) Give the eligibility criteria, and the sources and methods of selection of participants	6
Variables	7	Clearly define all outcomes, exposures, predictors, potential confounders, and effect modifiers. Give diagnostic criteria, if applicable	6-7
Data sources/ measurement	8*	For each variable of interest, give sources of data and details of methods of assessment (measurement). Describe comparability of assessment methods if there is more than one group	6-7
Bias	9	Describe any efforts to address potential sources of bias	*7*
Study size	10	Explain how the study size was arrived at	6-7
Quantitative variables	11	Explain how quantitative variables were handled in the analyses. If applicable, describe which groupings were chosen and why	6-7
Statistical methods	12	(*a*) Describe all statistical methods, including those used to control for confounding	6-7
		(*b*) Describe any methods used to examine subgroups and interactions	6-8
		(*c*) Explain how missing data were addressed	6-8
		(*d*) If applicable, describe analytical methods taking account of sampling strategy	6-8
		(*e*) Describe any sensitivity analyses	6-8
**Results**			

For peer review only

Participants	13*	(a) Report numbers of individuals at each stage of study—eg numbers potentially eligible, examined for eligibility, confirmed eligible, included in the study, completing follow-up, and analysed	9
		(b) Give reasons for non-participation at each stage	N/a
		(c) Consider use of a flow diagram	N/a
Descriptive data	14*	(a) Give characteristics of study participants (eg demographic, clinical, social) and information on exposures and potential confounders	
		(b) Indicate number of participants with missing data for each variable of interest	N/a
Outcome data	15*	Report numbers of outcome events or summary measures	9-10
Main results	16	(*a*) Give unadjusted estimates and, if applicable, confounder-adjusted estimates and their precision (eg, 95% confidence interval). Make clear which confounders were adjusted for and why they were included	9-10
		(*b*) Report category boundaries when continuous variables were categorized	9-10
		(*c*) If relevant, consider translating estimates of relative risk into absolute risk for a meaningful time period	NA
Other analyses	17	Report other analyses done—eg analyses of subgroups and interactions, and sensitivity analyses	9-10
**Discussion**			
Key results	18	Summarise key results with reference to study objectives	10-11
Limitations	19	Discuss limitations of the study, taking into account sources of potential bias or imprecision. Discuss both direction and magnitude of any potential bias	10-11
Interpretation	20	Give a cautious overall interpretation of results considering objectives, limitations, multiplicity of analyses, results from similar studies, and other relevant evidence	10-11
Generalisability	21	Discuss the generalisability (external validity) of the study results	10-11
**Other information**			
Funding	22	Give the source of funding and the role of the funders for the present study and, if applicable, for the original study on which the present article is based	14

*Give information separately for cases and controls in case-control studies and, if applicable, for exposed and unexposed groups in cohort and cross-sectional studies.

Note: An Explanation and Elaboration article discusses each checklist item and gives methodological background and published examples of transparent reporting. The STROBE checklist is best used in conjunction with this article (freely available on the Web sites of PLoS Medicine at http://www.plosmedicine.org/, Annals of Internal Medicine at http://www.annals.org/, and Epidemiology at http://www.epidem.com/). Information on the STROBE Initiative is available at www.strobe-statement.org.

For peer review only

BMJ Open

Are population trends in high-risk alcohol consumption in smokers associated with trends in quit attempts and quit success? A time series analysis

Journal:	*BMJ Open*
Manuscript ID	bmjopen-2019-034262.R1
Article Type:	Original research
Date Submitted by the Author:	06-Jan-2020

Complete List of Authors:	Beard, Emma; UCL,<br>Brown, Jamie; University College London, Psychology & Language Sciences<br>West, Robert; UCL<br>Michie, Susan; University College London, Centre for Outcomes Research and Effectivenes

**Primary Subject Heading**:	Addiction
Secondary Subject Heading:	Addiction, Epidemiology, Public health, Smoking and tobacco, Global health
Keywords:	time-series, tobacco, alcohol, high-risk drinking, quit attempts

SCHOLARONE™ Manuscripts

I, the Submitting Author has the right to grant and does grant on behalf of all authors of the Work (as defined in the below author licence), an exclusive licence and/or a non-exclusive licence for contributions from authors who are: i) UK Crown employees; ii) where BMJ has agreed a CC-BY licence shall apply, and/or iii) in accordance with the terms applicable for US Federal Government officers or employees acting as part of their official duties; on a worldwide, perpetual, irrevocable, royalty-free basis to BMJ Publishing Group Ltd ("BMJ") its licensees and where the relevant Journal is co-owned by BMJ to the co-owners of the Journal, to publish the Work in this journal and any other BMJ products and to exploit all rights, as set out in our licence.

The Submitting Author accepts and understands that any supply made under these terms is made by BMJ to the Submitting Author unless you are acting as an employee on behalf of your employer or a postgraduate student of an affiliated institution which is paying any applicable article publishing charge ("APC") for Open Access articles. Where the Submitting Author wishes to make the Work available on an Open Access basis (and intends to pay the relevant APC), the terms of reuse of such Open Access shall be governed by a Creative Commons licence – details of these licences and which Creative Commons licence will apply to this Work are set out in our licence referred to above.

Other than as permitted in any relevant BMJ Author's Self Archiving Policies, I confirm this Work has not been accepted for publication elsewhere, is not being considered for publication elsewhere and does not duplicate material already published. I confirm all authors consent to publication of this Work and authorise the granting of this licence.

For peer review only

Are population trends in high-risk alcohol consumption in smokers associated with trends in quit attempts and quit success? A time series analysis

Version 1

Dr Emma Beard[1]
[1] Research Department of Behavioural Science and Health, University College London, London 6BT, UK
e.beard@ucl.ac.uk

Dr Jamie Brown[1]
[1] Research Department of Behavioural Science and Health, University College London, London
jamie.brown@ucl.ac.uk

Professor Robert West[1]
[1] Research Department of Behavioural Science and Health, University College London, London
robert.west@ucl.ac.uk

Professor Susan Michie[2]
[2] Department of Clinical, Educational and Health Psychology, University College London, London
s.michie@ucl.ac.uk

For peer review only

To submit to: BMJ OPEN

Short title: Population trends in high-risk alcohol consumption among smokers

Word count: 2,440

Key words: time-series, tobacco, alcohol, high-risk drinking, quit attempts

Correspondence to: Emma Beard, Research Department of Behavioural Science and Health, University College London, WC1E 6BP. Email: e.beard@ucl.ac.uk. Tel: 02031083179

For peer review only - http://bmjopen.bmj.com/site/about/guidelines.xhtml

Abstract

Objectives: Monthly changes in the prevalence of high-risk drinking and smoking in England appear to be positively correlated. This study aimed to assess how far monthly changes in high-risk drinking were specifically associated with attempts to stop smoking and success of quit attempts.

Design: Data were used from the Alcohol and Smoking Toolkit Studies between April 2014 and June 2018. These involve monthly household face-to-face surveys of representative samples of ~1800 adults.

Setting: England

Participants: Data were aggregated on 17,560 past year smokers over the study period.

Primary and secondary outcome measures: ARIMAX modelling was used to assess the association over time between monthly prevalence of high-risk drinking among smokers and a) prevalence of attempts to quit smoking and b) prevalence of successful quit attempts in those attempting to quit. Bayes Factors (BF) were calculated to compare the null hypothesis with the hypothesis of an effect sufficiently large (β=0.6) to explain the established association between overall prevalence in smoking and high-risk drinking.

Results: No statistically significant associations were found between monthly changes in prevalence of high-risk drinking among smokers and attempts to quit smoking (β=0.156, 95%CI -0.079 to 0.391, p=0.194) or quit success (β=0.066, 95%CI -0.524 to 0.655, p=0.827). Bayes Factors (BF) indicated that the data were insensitive but suggested there is weak evidence for the null hypothesis in the case of both quit attempts (BF=0.80) and quit success (BF=0.53).

Conclusions: Monthly changes in prevalence of high-risk alcohol consumption in England are not clearly associated with changes in quit attempt or quit success rates.

Strengths and limitations of this study

- This is the first time series study to assess how far monthly changes in high-risk drinking are associated with attempts to stop smoking and success of quit attempts.

For peer review only

For peer review only - http://bmjopen.bmj.com/site/about/guidelines.xhtml

- This study uses a large representative sample of the population in England.
- In countries with weaker tobacco control different effects may be observed.
- Data are observational and so strong conclusions regarding cause and effect cannot be made.

For peer review only

For peer review only - http://bmjopen.bmj.com/site/about/guidelines.xhtml

Background

In England, around 15% of the population are smokers and 20% drink alcohol at high-risk levels, i.e. levels which are likely to cause harm (1, 2). Both are associated with a number of preventable conditions and appear to have an accumulative effect on the risk of mortality(3). The association between high-risk drinking and smoking has been well established at an individual level. High-risk drinkers are substantially more likely to smoke (4-8) and smokers who report starting a quit attempt also report lower alcohol consumption (9, 10). Attempts to quit smoking are also less successful among those with an alcohol use disorder (11-13). Such associations may arise by a number of mechanisms. For example, smokers drinking at high-risk levels may follow advice that it is important to restrict alcohol consumption when they quit(9, 14-16), alcohol and smoking appear to provide cues to lapses for the other and there may be pharmacological interactions between nicotine and alcohol (17-19). This is contrary to the popular notion of self-medication and reward seeking with people deprived of cigarettes compensating by increasing their use of alcohol (20).

It is important to identify whether similar patterns exist at a population level. An association in either direction could mean that policies that reduce smoking prevalence may have the added benefit of reducing high-risk drinking or vice versa. In England, since 2014 monthly data have been gathered on high-risk drinking, smoking status, attempts to quit smoking and quit success (21). Recently, we used these data to examine population-level associations over time between smoking and high-risk drinking and showed that monthly changes in prevalence of smoking in England were associated positively with prevalence of high-risk drinking. However, there were no significant associations between motivation to stop and motivation to reduce alcohol consumption, or attempts to quit smoking and attempts to reduce alcohol consumption (22). We found the combination of results surprising and suggested that the association with overall prevalence may be related to an unmeasured variable that accounted for the change in both smoking and high-risk drinking. Alternatively, the failure to find an overall association between motivation and attempts for each

For peer review only - http://bmjopen.bmj.com/site/about/guidelines.xhtml

behaviour may be an issue of power when focussing on the global association between subsamples that represented only a fifth of the overall sample.

This study attempted to resolve this apparent contradiction and explore the previously identified positive association between prevalence of smoking and prevalence of high-risk drinking. We relied on the assessment of trends between more specific outcomes expected to be more strongly related, if the identified association between the changes in the overall prevalence of smoking and high-drinking was causal. Specifically, we will assess whether changes in trends of excessive alcohol consumption among smokers are associated with trends in attempts to quit smoking and quit success.. If no association is found, this would support the conclusion of a third unmeasured variable associated with both smoking and high-risk drinking.

This study addressed the following research questions:

1. Is there an association in England between increases or decreases in monthly prevalence of high-risk drinking among smokers and attempts to quit smoking?
2. Is there an association in England between increases or decreases in monthly prevalence of high-risk drinking among smokers and quit success rates?

Methods

Study design

Data were used from the Smoking and Alcohol Toolkit Studies (STS and ATS) collected between April 2014 and June 2018. The STS and ATS are ongoing studies that involve a series of monthly cross-sectional household, face-to-face, computer assisted surveys of representative samples of ~1800 adults in England aged 16+. Thus, the same participants take part in both surveys. The respondents are recruited using a type of random location sampling, which is a hybrid between

For peer review only - http://bmjopen.bmj.com/site/about/guidelines.xhtml

random probability and simple quota sampling. England is first split into over 170000 'Output Areas', comprising of approximately 300 households. These areas are then stratified according to ACORN characteristics and geographic region (http://www.caci.co.uk/acorn/) and are randomly allocated to interviewers. Interviewers visit households within their allocated locality starting at a random point in the area. One member per household, chosen based on who the interviewer judge would best fulfil their quota requirements, is interviewed until interviewers achieve local quotas designed to minimise differences in the probability of participation. Participants appear to be representative of the population in England, having similar socio-demographic composition and smoking characteristics to large national surveys based on probability samples such as the Health Survey for England (23), while drinking characteristics also appear similar at a regional level to other national surveys (24). For further details see: www.smokinginengland.info and www.alcoholinengland.info and the published protocol (21, 23).

Participants

Data were collected on 88,122 participants over the study period. Of these, 19.94% (95%CI 19.67 to 20.20 n=17,560) reported that they had smoked in the past year. Forty-seven percent of past year smokers (n=8097) were male, 18.9% (n=3272) were aged 16-24, 19.7% (n=3416) were aged 25 to 34, 16.2% (n=2804) were aged 35 to 44, 17.0% (n=2946) were aged 45 to 54, 14.6% (n=2521) were aged 55 to 64 and 13.7% (n=2371) were aged 65+. Finally, 59.4% (n=10286) were in manual occupations. Data from these participants were aggregated monthly and this forms the basis of the sample in this paper.

Measures

Participants were asked whether they smoked or had smoked cigarettes (including hand-rolled) daily or non-daily in the past year and to complete the Alcohol Use Disorders Identification Test (AUDIT) (25). The AUDIT identifies people who could be classed as dependent, harmful or

For peer review only - http://bmjopen.bmj.com/site/about/guidelines.xhtml

hazardous drinkers and has demonstrated validity, high internal consistency and good test-retest reliability across gender, age and cultures (26-29). Those scoring 8 or more were classed as high-risk drinkers. This is a common threshold for high-risk drinkers (27, 30-32). The prevalence of high-risk alcohol consumption among smokers in each month was obtained by counting the number smokers reporting an AUDIT score greater than or equal to 8.

Past year smokers were then asked:

1. "How many serious attempts to stop smoking have you made in the last 12 months? By serious attempt I mean you decided that you would try to make sure you never smoked again. Please include any attempt that you are currently making and please include any successful attempt made within the last year".
2. "How long did your most recent serious quit attempt last before you went back to smoking?"

The prevalence of quit attempts in monthly were calculated as the number of respondents who reported having made one or more quit attempts in the past 12 months divided by the number of past year smokers. The success rate in each quarter was calculated as the number of respondents reporting that they were still not smoking divided by the number reporting having made a quit attempt.

Analysis

The analysis plan, data and syntax were preregistered on the Open Science Framework (https://osf.io/384gx/). Cases with missing data on either smoking or drinking variables were classified as missing in calculating the prevalence figures: smoking status (n=55; %=0.06), high-risk drinking status among smokers (n=202; %=1.17) and quit attempts among smokers (n=562; %=3.24). All data were analysed in R studio.

For peer review only - http://bmjopen.bmj.com/site/about/guidelines.xhtml

Data were weighted (see (23) for further details) to match the population in England and analysed using Autoregressive Integrated Moving Average with Exogeneous Input (ARIMAX) modelling to assess the association between prevalence of high risk drinking among smokers and 1) prevalence of attempts to quit smoking and 2) prevalence of successful attempts to quit smoking among those having made a quit attempt. ARIMAX is an extension of autoregressive integrated moving average analysis (ARIMA), which produces forecasts based upon prior values in the time series (Autoregressive terms; AR) and the errors made by previous predictions (Moving Average terms; MA). We followed a standard ARIMAX modelling approach (33).

The ARIMAX assumption of weak exogeneity was met: past prevalence of quit attempts ($p=0.747$) and quit success ($p=0.999$) did not statistically predict future prevalence of high-risk drinking among smokers. No outliers were identified in any of the series using an approach based on that described by Chen and Liu(34, 35). To stabilise the variance the series were log-transformed. The Augmented Dickey-Fuller test and visual inspection of the plots indicated that first order differencing was required for both time series. First order differencing involves calculating the change between one observation and the next. No additional seasonal differencing was required (36).

The autocorrelation and partial autocorrelation functions were examined to determine the non-seasonal MA and AR terms. These suggested an ARIMAX(0,1,1) model for the time series predicting both prevalence of quit attempts and prevalence of quit success. This was confirmed by comparing models with different specifications using the AIC. To identify the most appropriate transfer function for the continuous explanatory variables the sample cross-correlation function was checked and models with varying distributed lags compared using the Akaike Information Criterion. This suggested a lag of 0 when predicting the prevalence of quit attempts and predicting

For peer review only - http://bmjopen.bmj.com/site/about/guidelines.xhtml

the prevalence of quit success, thus only current values and not lagged (past period) values of the input series were used to predict current values of the output series. In our previous study, prevalence of smoking was found to be associated with high-risk drinking with a distributed lag of 2 (22). Thus, additional sensitivity analyses were run with the output series lagged by an order of 2 i.e. the time base was shifted back by 2 months.

The Ljung-Box test for white noise showed that the residuals for both fitted models were free of serial correlation. A number of additional model checks were also made. First, the autocorrelation terms included in the model were checked for their statistical significance. Secondly, it was determined whether the model residuals were normally distributed, random and independent. Finally, that the inclusion of the MA term conformed to the bounds of invertibility i.e. its value was <1 (33, 34).

Bayes factors (BFs) were derived for non-significant findings using an online calculator to disentangle whether there is evidence for the null hypothesis of no effect (BF <1/3rd) or the data are insensitive (BF between 1/3rd and 3)(37, 38). A half-normal distribution was assumed with a percentage change in the outcomes of interest for every percentage increase in the input series of 0.6%. This is on the basis of a previous study showing that smokers who had made a quit attempt were around 40% less likely to report that they were high risk drinkers (9). Strengthening the Reporting of Observational Studies in Epidemiology (STROBE) guidelines for the reporting of observational studies were followed throughout (39).

Patient involvement

No patients were involved in setting the research question or the outcome measures, nor were they involved in developing plans for recruitment, design, or implementation of the study. No patients

For peer review only - http://bmjopen.bmj.com/site/about/guidelines.xhtml

were asked to advise on interpretation or writing up of results. There are no plans to disseminate the results of the research directly to study participants or any specific patient community.

Results

Figure 1 shows the raw time series data from 2014 to 2018. Prevalence of high-risk drinking among smokers declined from 26.9% (95% CI 22.34 to 32.03) in 2014 to 23.7% (95% CI 19.26 to 28.87) in June 2018. Attempts to quit smoking also declined from 38.1% (95%CI 32.86 to 43.66) to 28.5% (95%CI 23.60 to 33.90) and quit success from 19.6% (95%CI 13.22 to 27.87) to 9.4% (95%CI 4.51 to 17.95) in June 2018.

Tables 1 shows the results of the ARIMAX models assessing the association between prevalence of high-risk drinking among smokers and (1) quit attempts and (2) quit success. The findings were inconclusive as to whether any associations were present. BFs suggested that there is anecdotal evidence for the null hypothesis that prevalence of high-risk drinking among smokers is not associated with prevalence of quit attempts and prevalence of quit success. Findings were similar when a 2 month back shifted lag was used for prevalence of quit attempts and quit success.

Discussion

To our knowledge, this is the first empirical study to estimate the population association between high-risk drinking among smokers and attempts to quit smoking and the success of those attempts. There was weak evidence that there was no substantial association between changes in the prevalence of high-risk drinking and quit attempts and quit success.

These findings appear to be at odds with individual level studies which suggest that smokers with an alcohol use disorder are less likely to attempt and succeed in stopping smoking (12, 13). Alcohol consumption during attempts at smoking cessation is also associated with a greater risk of relapse (14). As a result, smokers are often advised to lower their alcohol consumption when they attempt

For peer review only - http://bmjopen.bmj.com/site/about/guidelines.xhtml

For peer review only

to quit smoking(9). Of course, it remains plausible that high-risk drinking among smokers may still be associated with a small effect on mean population prevalence of quit attempts and their success, but it was not possible to detect this in the current study. An association may also be masked by factors impacting at a population level which were not accounted for in the current study. Although we are unaware of any major population-level interventions or other events during the study period which may have affected the associations under investigation, we cannot rule out residual confounding. There may also be some statistical bias due to the loss of power and sensitivity that comes with aggregating data. Prevalence of high-risk drinking among smokers will also be somewhat noisier than if prevalence was also assessed among non-smokers, given the smaller sample size involved in estimation.

These findings suggest that the previously identified positive association between prevalence of smoking and prevalence of high-risk drinking is unlikely to be causal, whereby smokers attempting to quit, and those succeeding, also reduce their alcohol intake (22). Although it remains possible that use of alcohol by smokers impacts on other key indices including longer term abstinence, the small proportion of smokers who relapse long term (i.e. after a year) could not account for the size of association noted. It may instead be that overall prevalence is related to an unmeasured variable, perhaps economic factors and sociocultural events, that account for the change in both smoking and high-risk drinking. For example, in recent years taxation on cigarettes and alcohol have increased linearly, driving down sales of both (40, 41). There have also been substantial fluctuations in average household income since 2013, which have been shown to independently affect smoking and alcohol consumption (42-44). Sporting events such as the Olympics may also concurrently increase alcohol and tobacco intake as they are celebratory occasions. Mass media campaigns may also play role, simultaneously promoting attempts to quit smoking and the adoption of a healthier lifestyle by reducing alcohol intake (45).

For peer review only - http://bmjopen.bmj.com/site/about/guidelines.xhtml

A strength of this study is the use of a large representative sample of the population in England. Several limitations need to be considered. First, the ATS required participants to recall their alcohol consumption and attempts to quit smoking which is likely to have been somewhat inaccurate due to recall bias and social desirability. For example, it has been found that a large proportion of unsuccessful quit attempts fail to be reported, particularly if they only last a short time or occurred longer ago(46). However, social pressure in population surveys tends to be low and so it is generally considered acceptable to rely on self-report data(47). Second, these findings may not generalise to other countries. England has a strong tobacco-control climate. In countries with weaker tobacco control or different alcohol control policies, different effects may be observed. Thirdly, this paper did not consider the impact of changes in excessive alcohol consumption prevalence on the length of quit success, being defined as having made a quit in attempt in the last 12 months and still reporting not smoking. This will be an important area for future research as more data are accumulated to provide adequate power. Finally, although there can be no individual-level confounding in population trend data there is a possibility of population-level confounding, such as introduction of policies that may affect quitting rates. However, we were unable to identify any such population policies occurring during the study period that may have confounded the results.

Conclusion

These findings suggest that the previously identified positive association between prevalence of smoking and prevalence of high-risk drinking is unlikely to be causal, whereby smokers attempting to quit, and those succeeding, also reduce their alcohol intake. Instead, it may be that overall prevalence is related to an unmeasured third variable such as economic factors and sociocultural events.

For peer review only

For peer review only - http://bmjopen.bmj.com/site/about/guidelines.xhtml

List of abbreviations

ARIMAX - autoregressive integrated moving average with exogenous input
BF – Bayes Factor
ATS – Alcohol Toolkit Study
STS – Smoking Toolkit Study

For peer review only

For peer review only - http://bmjopen.bmj.com/site/about/guidelines.xhtml

Table 1: Estimated percentage point changes in proportion of quit attempts and proportion of quitters who met criteria for quit success during the study period, based on autoregressive integrated moving average with exogenous input (ARIMAX) models

		Output series					
		Quit attempts[1]			Quit success[2]		
		Percentage change per 1% change in the exposure	95%CI	p	Percentage change per 1% change in the exposure	95%CI	p
Input series	**Model 1:** High-risk drinking among smokers (no backward lag of the output series)	0.156	-0.079 to 0.391	0.194	0.066	-0.524 to 0.655	0.827
	**Model 2:** High-risk drinking among smokers (two month backward lag of the output series)	0.065	-0.183 to 0.313	0.608	0.134	-0.469 to 0.736	0.663
	**Bayes Factor**						
	Model 1	0.80			0.53		
	Model 2	0.33			0.64		

Figure 1: Prevalence of a) high-risk drinking; b) attempts to quit smoking and c) quit success

Declarations

Ethical approval and consent to participate

Ethics approval for the Smoking Toolkit Survey (STS) was originally granted by the UCL Ethics Committee (ID 0498/001) and approval for the ATS was granted by the same committee as an extension of the STS (ID 2808/005). In accordance with our ethical approval, all respondents were given a written information sheet about the study, and provided informed verbal consent

Availability of data and material

The analysis plan, data and syntax were preregistered on the Open Science Framework (https://osf.io/384gx/).

Funding

The Smoking Toolkit Study is currently primarily funded by Cancer Research UK (C1417/A14135; C36048/A11654; C44576/A19501) and has previously also been funded by Pfizer, GSK, and the Department of Health. The ATS is currently funded by the NIHR School for Public Health Research (SPHR) (SPHR-SWP-ALC-WP5). SPHR is a partnership between the Universities of Sheffield; Bristol; Cambridge; Exeter; UCL; The London School for Hygiene and Tropical Medicine; the LiLaC collaboration between the Universities of Liverpool and Lancaster and Fuse; The Centre for Translational Research in Public Health, a collaboration between Newcastle, Durham, Northumbria, Sunderland and Teesside Universities. The views expressed are those of the authors(s) and not necessarily those of the NHS, NIHR, or Department of Health. No funders had any involvement in the design of the study, the analysis or interpretation of the data, the writing of the report, or the decision to submit the paper for publication. JB's post is funded CRUK

For peer review only

For peer review only - http://bmjopen.bmj.com/site/about/guidelines.xhtml

(C1417/A14135). RW is funded by Cancer Research UK (C1417/A14135). EB is funded by the NIHR SPHR (SPHR-SWP-ALC-WP5) and CRUK also provide support (C1417/A14135).

Competing interests

RW undertakes consultancy and research for and receives travel funds and hospitality from manufacturers of smoking cessation medications. RW salary is funded by Cancer Research UK. SM receives support from Cancer Research UK and the National Institute for Health Research (NIHR)'s School for Public Health Research (SPHR). EB and JB have received unrestricted research funding from Pfizer. PM's research is funded by a variety of governmental funding agencies including UKRI and NIHR.

Authors' contributions

EB, JB, SM and RW wrote the first draft of the manuscript and conducted the analysis. All other authors commented on this draft and contributed to the final version. All authors read and approved the final manuscript.

For peer review only

For peer review only - http://bmjopen.bmj.com/site/about/guidelines.xhtml

References

1. Statistics OfN. Adult smoking habits in the UK: 2017. 2018.
2. Health and Social Care Information Centre. Statistics on Alcohol, England 2016 2016 [Available from: https://www.gov.uk/government/statistics/statistics-on-alcohol-england-2016.
3. Kvaavik E, Batty GD, Ursin G, Huxley R, Gale CR. Influence of individual and combined health behaviors on total and cause-specific mortality in men and women: the United Kingdom health and lifestyle survey. Archives of internal medicine. 2010;170(8):711-8.
4. Lasser K, Boyd JW, Woolhandler S, Himmelstein DU, McCormick D, Bor DH. Smoking and mental illness: a population-based prevalence study. JAMA. 2000;284.
5. Falk DE, Yi HY, Hiller-Sturmhofel S. An epidemiologic analysis of co-occurring alcohol and tobacco use and disorders: findings from the National Epidemiologic Survey on Alcohol and Related Conditions. Alcohol Res Health. 2006;29.
6. Kalman D, Morissette SB, George TP. Co-morbidity of smoking in patients with psychiatric and substance use disorders. Am J Addict. 2005;14.
7. Kessler RC, Chiu WT, Demler O, Merikangas KR, Walters EE. Prevalence, severity, and comorbidity of 12-month DSM-IV disorders in the National Comorbidity Survey Replication. Arch Gen Psychiatry. 2005;62.
8. Hasin DS, Stinson FS, Ogburn E, Grant BF. Prevalence, correlates, disability, and comorbidity of DSM-IV alcohol abuse and dependence in the United States: results from the National Epidemiologic Survey on Alcohol and Related Conditions. Arch Gen Psychiatry. 2007;64.
9. Brown J, West R, Beard E, Brennan A, Drummond C, Gillespie D, et al. Are recent attempts to quit smoking associated with reduced drinking in England? A cross-sectional population survey. BMC Public Health. 2016;16(1):1-7.
10. Przulj D, Hajek P, Snuggs S, McRobbie H. Changes in Alcohol Consumption During a Stop-Smoking Attempt and Differences Between Smokers Using Nicotine Replacement and Smokers Using Varenicline. Nicotine Tob Res. 2018;20(5):583-8.
11. Hughes JR, Kalman D. Do smokers with alcohol problems have more difficulty quitting? Drug Alcohol Depend. 2006;82.
12. Weinberger AH, Pilver CE, Hoff RA, Mazure CM, McKee SA. Changes in smoking for adults with and without alcohol and drug use disorders: longitudinal evaluation in the U.S. population. Am J Drug Alcohol Abuse. 2013;39.
13. Leeman RF, McKee SA, Toll BA, Krishnan-Sarin S, Cooney JL, Makuch RW, et al. Risk factors for treatment failure in smokers: relationship to alcohol use and to lifetime history of an alcohol use disorder. Nicotine Tob Res. 2008;10.
14. Kahler CW, Spillane NS, Metrik J. Alcohol use and initial smoking lapses among heavy drinkers in smoking cessation treatment. Nicotine Tob Res. 2010;12.
15. Gulliver SB, Kamholz BW, Helstrom AW. Smoking cessation and alcohol abstinence: what do the data tell us? Alcohol Res Health. 2006;29.
16. Friend KB, Pagano ME. Smoking cessation and alcohol consumption in individuals in treatment for alcohol use disorders. J Addict Dis. 2005;24.
17. Rose JE, Brauer LH, Behm FM, Cramblett M, Calkins K, Lawhon D. Psychopharmacological interactions between nicotine and ethanol. Nicotine & Tobacco Research. 2004;6(1):133-44.
18. Field M, Mogg K, Bradley BP. Alcohol increases cognitive biases for smoking cues in smokers. Psychopharmacology. 2005;180(1):63-72.
19. Verplaetse TL, McKee SA. An overview of alcohol and tobacco/nicotine interactions in the human laboratory. The American journal of drug and alcohol abuse. 2017;43(2):186-96.
20. Blume AW, Schmaling KB, Marlatt GA. Revisiting the self-medication hypothesis from a behavioral perspective. Cognitive and Behavioral Practice. 2000;7(4):379-84.
21. Beard E, Brown J, West R, Acton C, Brennan A, Drummond C, et al. Protocol for a national monthly survey of alcohol use in England with 6-month follow-up:'The Alcohol Toolkit Study'. BMC public health. 2015;15(1):230.

For peer review only - http://bmjopen.bmj.com/site/about/guidelines.xhtml

22. Beard E, West R, Michie S, Brown J. Association between smoking and alcohol-related behaviours: a time–series analysis of population trends in England. Addiction (Abingdon, England). 2017;112(10):1832-41.
23. Fidler JA, Shahab L, West O, Jarvis MJ, McEwen A, Stapleton JA, et al. 'The smoking toolkit study': a national study of smoking and smoking cessation in England. BMC Public Health. 2011;11(1):479.
24. Beard E, Brown J, West R, Angus C, Kaner E, Michie S. Healthier central England or North–South divide? Analysis of national survey data on smoking and high-risk drinking. BMJ Open. 2017;7(3).
25. Casswell S, Meier P, MacKintosh AM, Brown A, Hastings G, Thamarangsi T, et al. The International Alcohol Control (IAC) Study—Evaluating the Impact of Alcohol Policies. Alcoholism: Clinical and Experimental Research. 2012;36(8):1462-7.
26. Saunders JB, Aasland OG, Amundsen A, Grant M. Alcohol consumption and related problems among primary health care patients: WHO collaborative project on early detection of persons with harmful alcohol consumption--I. Addiction (Abingdon, England). 1993;88(3):349-62.
27. Saunders JB, Aasland OG, Babor TF, de la Fuente JR, Grant M. Development of the Alcohol Use Disorders Identification Test (AUDIT): WHO Collaborative Project on Early Detection of Persons with Harmful Alcohol Consumption--II. Addiction (Abingdon, England). 1993;88(6):791-804.
28. Allen JP, Litten RZ, Fertig JB, Babor T. A Review of Research on the Alcohol Use Disorders Identification Test (AUDIT). Alcoholism: Clinical and Experimental Research. 1997;21(4):613-9.
29. Hays R, Merz J, Nicholas R. Response burden, reliability, and validity of the CAGE, Short MAST, and AUDIT alcohol screening measures. Behavior Research Methods, Instruments, & Computers. 1995;27(2):277-80.
30. Caviness CM, Hatgis C, Anderson BJ, Rosengard C, Kiene SM, Friedmann PD, et al. Three Brief Alcohol Screens for Detecting Hazardous Drinking in Incarcerated Women. Journal of Studies on Alcohol and Drugs. 2009;70(1):50-4.
31. National Institute on Alcohol Abuse and Alcoholism. Helping Patients Who Drink Too Much: A Clinician's Guide. Bethesda, MD: National Institute on Alcohol Abuse and Alcoholism; 2007.
32. Kokotailo PK, Egan J, Gangnon R, Brown D, Mundt M, Fleming M. Validity of the alcohol use disorders identification test in college students. Alcoholism: Clinical and Experimental Research. 2004;28(6):914-20.
33. Box GE, Jenkins GM, Reinsel GC. Time series analysis: forecasting and control: John Wiley & Sons; 2011.
34. Chen C, Liu L-M. Joint estimation of model parameters and outlier effects in time series. Journal of the American Statistical Association. 1993;88(421):284-97.
35. López-de-Lacalle J. tsoutliers R Package for Automatic Detection of Outliers in Time Series.
36. Dickey DA, Fuller WA. Distribution of the estimators for autoregressive time series with a unit root. Journal of the American statistical association. 1979;74(366a):427-31.
37. Dienes Z. Using Bayes to get the most out of non-significant results. Frontiers in Psychology. 2014;5(781).
38. Beard E, Dienes Z, Muirhead C, West R. Using Bayes factors for testing hypotheses about intervention effectiveness in addictions research. Addiction (Abingdon, England). 2016;111(12):2230-47.
39. Von Elm E, Altman DG, Egger M, Pocock SJ, Gøtzsche PC, Vandenbroucke JP, et al. The Strengthening the Reporting of Observational Studies in Epidemiology (STROBE) statement: guidelines for reporting observational studies. PLoS medicine. 2007;4(10):e296.

For peer review only - http://bmjopen.bmj.com/site/about/guidelines.xhtml

40. Institute of Alcohol Studies. How have UK alcohol taxes changed over time? 2017 [Available from: http://www.ias.org.uk/Alcohol-knowledge-centre/Price/Factsheets/How-have-UK-alcohol-taxes-changed-over-time.aspx.
41. ASH. Timeline of changes in tobacco taxation in the UK from 1993 to the present 2017 [Available from: http://ash.org.uk/information-and-resources/taxation-illicit-trade/taxation/ash-analysis-of-tobacco-tax-increases-in-the-united-kingdom/.
42. Cerdá M, Johnson-Lawrence V, Galea S. Lifetime income patterns and alcohol consumption: Investigating the association between long- and short-term income trajectories and drinking. Social science & medicine (1982). 2011;73(8):1178-85.
43. Perelman J, Alves J, Pfoertner TK, Moor I, Federico B, Kuipers MAG, et al. The association between personal income and smoking among adolescents: a study in six European cities. Addiction (Abingdon, England). 2017;112(12):2248-56.
44. ONS. Nowcasting household income in the UK: financial year ending 2017. 2018.
45. Wakefield MA, Loken B, Hornik RC. Use of mass media campaigns to change health behaviour. The Lancet. 2010;376(9748):1261-71.
46. Berg CJ, An LC, Kirch M, Guo H, Thomas JL, Patten CA, et al. Failure to report attempts to quit smoking. Addictive behaviors. 2010;35(10):900-4.
47. Wong SL, Shields M, Leatherdale S, Malaison E, Hammond D. Assessment of validity of self-reported smoking status. Health reports. 2012;23(1):D1.

For peer review only

For peer review only - http://bmjopen.bmj.com/site/about/guidelines.xhtml

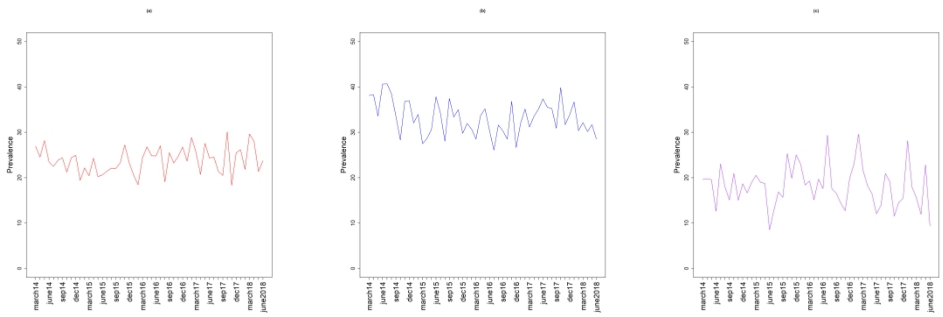

762x254mm (300 x 300 DPI)

STROBE 2007 (v4) Statement—Checklist of items that should be included in reports of *cross-sectional studies*

**Section/Topic**	**Item #**	**Recommendation**	**Reported on page #**
**Title and abstract**	1	(*a*) Indicate the study's design with a commonly used term in the title or the abstract	1
		(*b*) Provide in the abstract an informative and balanced summary of what was done and what was found	2
**Introduction**			
Background/rationale	2	Explain the scientific background and rationale for the investigation being reported	4-5
Objectives	3	State specific objectives, including any prespecified hypotheses	5
**Methods**			
Study design	4	Present key elements of study design early in the paper	6
Setting	5	Describe the setting, locations, and relevant dates, including periods of recruitment, exposure, follow-up, and data collection	6
Participants	6	(*a*) Give the eligibility criteria, and the sources and methods of selection of participants	6
Variables	7	Clearly define all outcomes, exposures, predictors, potential confounders, and effect modifiers. Give diagnostic criteria, if applicable	6-7
Data sources/ measurement	8*	For each variable of interest, give sources of data and details of methods of assessment (measurement). Describe comparability of assessment methods if there is more than one group	6-7
Bias	9	Describe any efforts to address potential sources of bias	*7*
Study size	10	Explain how the study size was arrived at	6-7
Quantitative variables	11	Explain how quantitative variables were handled in the analyses. If applicable, describe which groupings were chosen and why	6-7
Statistical methods	12	(*a*) Describe all statistical methods, including those used to control for confounding	6-7
		(*b*) Describe any methods used to examine subgroups and interactions	6-8
		(*c*) Explain how missing data were addressed	6-8
		(*d*) If applicable, describe analytical methods taking account of sampling strategy	6-8
		(*e*) Describe any sensitivity analyses	6-8
**Results**			

For peer review only

Participants	13*	(a) Report numbers of individuals at each stage of study—eg numbers potentially eligible, examined for eligibility, confirmed eligible, included in the study, completing follow-up, and analysed	9
		(b) Give reasons for non-participation at each stage	N/a
		(c) Consider use of a flow diagram	N/a
Descriptive data	14*	(a) Give characteristics of study participants (eg demographic, clinical, social) and information on exposures and potential confounders	
		(b) Indicate number of participants with missing data for each variable of interest	N/a
Outcome data	15*	Report numbers of outcome events or summary measures	9-10
Main results	16	(*a*) Give unadjusted estimates and, if applicable, confounder-adjusted estimates and their precision (eg, 95% confidence interval). Make clear which confounders were adjusted for and why they were included	9-10
		(*b*) Report category boundaries when continuous variables were categorized	9-10
		(*c*) If relevant, consider translating estimates of relative risk into absolute risk for a meaningful time period	NA
Other analyses	17	Report other analyses done—eg analyses of subgroups and interactions, and sensitivity analyses	9-10
**Discussion**			
Key results	18	Summarise key results with reference to study objectives	10-11
Limitations	19	Discuss limitations of the study, taking into account sources of potential bias or imprecision. Discuss both direction and magnitude of any potential bias	10-11
Interpretation	20	Give a cautious overall interpretation of results considering objectives, limitations, multiplicity of analyses, results from similar studies, and other relevant evidence	10-11
Generalisability	21	Discuss the generalisability (external validity) of the study results	10-11
**Other information**			
Funding	22	Give the source of funding and the role of the funders for the present study and, if applicable, for the original study on which the present article is based	14

*Give information separately for cases and controls in case-control studies and, if applicable, for exposed and unexposed groups in cohort and cross-sectional studies.

Note: An Explanation and Elaboration article discusses each checklist item and gives methodological background and published examples of transparent reporting. The STROBE checklist is best used in conjunction with this article (freely available on the Web sites of PLoS Medicine at http://www.plosmedicine.org/, Annals of Internal Medicine at http://www.annals.org/, and Epidemiology at http://www.epidem.com/). Information on the STROBE Initiative is available at www.strobe-statement.org.

For peer review only

BMJ Open

Are population trends in high-risk alcohol consumption in smokers associated with trends in quit attempts and quit success? A time series analysis

Journal:	*BMJ Open*
Manuscript ID	bmjopen-2019-034262.R2
Article Type:	Original research
Date Submitted by the Author:	18-Feb-2020
Complete List of Authors:	Beard, Emma; UCL,<br>Brown, Jamie; University College London, Psychology & Language Sciences<br>West, Robert; UCL<br>Michie, Susan; University College London, Centre for Outcomes Research and Effectivenes
**Primary Subject Heading**:	Addiction
Secondary Subject Heading:	Addiction, Epidemiology, Public health, Smoking and tobacco, Global health
Keywords:	time-series, tobacco, alcohol, high-risk drinking, quit attempts

SCHOLARONE™
Manuscripts

I, the Submitting Author has the right to grant and does grant on behalf of all authors of the Work (as defined in the below author licence), an exclusive licence and/or a non-exclusive licence for contributions from authors who are: i) UK Crown employees; ii) where BMJ has agreed a CC-BY licence shall apply, and/or iii) in accordance with the terms applicable for US Federal Government officers or employees acting as part of their official duties; on a worldwide, perpetual, irrevocable, royalty-free basis to BMJ Publishing Group Ltd ("BMJ") its licensees and where the relevant Journal is co-owned by BMJ to the co-owners of the Journal, to publish the Work in this journal and any other BMJ products and to exploit all rights, as set out in our licence.

The Submitting Author accepts and understands that any supply made under these terms is made by BMJ to the Submitting Author unless you are acting as an employee on behalf of your employer or a postgraduate student of an affiliated institution which is paying any applicable article publishing charge ("APC") for Open Access articles. Where the Submitting Author wishes to make the Work available on an Open Access basis (and intends to pay the relevant APC), the terms of reuse of such Open Access shall be governed by a Creative Commons licence – details of these licences and which Creative Commons licence will apply to this Work are set out in our licence referred to above.

Other than as permitted in any relevant BMJ Author's Self Archiving Policies, I confirm this Work has not been accepted for publication elsewhere, is not being considered for publication elsewhere and does not duplicate material already published. I confirm all authors consent to publication of this Work and authorise the granting of this licence.

For peer review only

Are population trends in high-risk alcohol consumption in smokers associated with trends in quit attempts and quit success? A time series analysis

Version 1

Dr Emma Beard[1]
[1] Research Department of Behavioural Science and Health, University College London, London 6BT, UK
e.beard@ucl.ac.uk

Professor Jamie Brown[1]
[1] Research Department of Behavioural Science and Health, University College London, London
jamie.brown@ucl.ac.uk

Professor Robert West[1]
[1] Research Department of Behavioural Science and Health, University College London, London
robert.west@ucl.ac.uk

Professor Susan Michie[2]
[2] Department of Clinical, Educational and Health Psychology, University College London, London
s.michie@ucl.ac.uk

To submit to: BMJ OPEN

Short title: Population trends in high-risk alcohol consumption among smokers

Word count: 2,440

Key words: time-series, tobacco, alcohol, high-risk drinking, quit attempts

Correspondence to: Emma Beard, Research Department of Behavioural Science and Health, University College London, WC1E 6BP. Email: e.beard@ucl.ac.uk. Tel: 02031083179

For peer review only - http://bmjopen.bmj.com/site/about/guidelines.xhtml

Abstract

Objectives: Monthly changes in the prevalence of high-risk drinking and smoking in England appear to be positively correlated. This study aimed to assess how far monthly changes in high-risk drinking were specifically associated with attempts to stop smoking and success of quit attempts.

Design: Data were used from the Alcohol and Smoking Toolkit Studies between April 2014 and June 2018. These involve monthly household face-to-face surveys of representative samples of ~1800 adults.

Setting: England

Participants: Data were aggregated on 17,560 past year smokers over the study period.

Primary and secondary outcome measures: ARIMAX modelling was used to assess the association over time between monthly prevalence of high-risk drinking among smokers and a) prevalence of attempts to quit smoking and b) prevalence of successful quit attempts in those attempting to quit. Bayes Factors (BF) were calculated to compare the null hypothesis with the hypothesis of an effect sufficiently large (β=0.6) to explain the established association between overall prevalence in smoking and high-risk drinking.

Results: No statistically significant associations were found between monthly changes in prevalence of high-risk drinking among smokers and attempts to quit smoking (β=0.156, 95%CI -0.079 to 0.391, p=0.194) or quit success (β=0.066, 95%CI -0.524 to 0.655, p=0.827). Bayes Factors (BF) indicated that the data were insensitive but suggested there is weak evidence for the null hypothesis in the case of both quit attempts (BF=0.80) and quit success (BF=0.53).

Conclusions: Monthly changes in prevalence of high-risk alcohol consumption in England are not clearly associated with changes in quit attempt or quit success rates.

Strengths and limitations of this study

- This is the first time series study to assess how far monthly changes in high-risk drinking are associated with attempts to stop smoking and success of quit attempts.

For peer review only

For peer review only - http://bmjopen.bmj.com/site/about/guidelines.xhtml

- This study uses a large representative sample of the population in England.
- In countries with weaker tobacco control different effects may be observed.
- Data are observational and so strong conclusions regarding cause and effect cannot be made.

For peer review only

For peer review only - http://bmjopen.bmj.com/site/about/guidelines.xhtml

Background

In England, around 15% of the population are smokers and 20% drink alcohol at high-risk levels, i.e. levels which are likely to cause harm (1, 2). Both are associated with a number of preventable conditions and appear to have an accumulative effect on the risk of mortality(3). The association between high-risk drinking and smoking has been well established at an individual level. High-risk drinkers are substantially more likely to smoke (4-8) and smokers who report starting a quit attempt also report lower alcohol consumption (9, 10). Attempts to quit smoking are also less successful among those with an alcohol use disorder (11-13). Such associations may arise by a number of mechanisms. For example, smokers drinking at high-risk levels may follow advice that it is important to restrict alcohol consumption when they quit(9, 14-16), alcohol and smoking appear to provide cues to lapses for the other and there may be pharmacological interactions between nicotine and alcohol (17-19). This is contrary to the popular notion of self-medication and reward seeking with people deprived of cigarettes compensating by increasing their use of alcohol (20).

It is important to identify whether similar patterns exist at a population level. An association in either direction could mean that policies that reduce smoking prevalence may have the added benefit of reducing high-risk drinking or vice versa. In England, since 2014 monthly data have been gathered on high-risk drinking, smoking status, attempts to quit smoking and quit success (21). Recently, we used these data to examine population-level associations over time between smoking and high-risk drinking and showed that monthly changes in prevalence of smoking in England were associated positively with prevalence of high-risk drinking. However, there were no significant associations between motivation to stop and motivation to reduce alcohol consumption, or attempts to quit smoking and attempts to reduce alcohol consumption (22). We found the combination of results surprising and suggested that the association with overall prevalence may be related to an unmeasured variable that accounted for the change in both smoking and high-risk drinking. Alternatively, the failure to find an overall association between motivation and attempts for each

For peer review only - http://bmjopen.bmj.com/site/about/guidelines.xhtml

behaviour may be an issue of power when focussing on the global association between subsamples that represented only a fifth of the overall sample.

This study attempted to resolve this apparent contradiction and explore the previously identified positive association between prevalence of smoking and prevalence of high-risk drinking. We relied on the assessment of trends between more specific outcomes expected to be more strongly related, if the identified association between the changes in the overall prevalence of smoking and high-drinking was causal. Specifically, we will assess whether changes in trends of excessive alcohol consumption among smokers are associated with trends in attempts to quit smoking and quit success.. If no association is found, this would support the conclusion of a third unmeasured variable associated with both smoking and high-risk drinking.

This study addressed the following research questions:

1. Is there an association in England between increases or decreases in monthly prevalence of high-risk drinking among smokers and attempts to quit smoking?
2. Is there an association in England between increases or decreases in monthly prevalence of high-risk drinking among smokers and quit success rates?

Methods

Study design

Data were used from the Smoking and Alcohol Toolkit Studies (STS and ATS) collected between April 2014 and June 2018. The STS and ATS are ongoing studies that involve a series of monthly cross-sectional household, face-to-face, computer assisted surveys of representative samples of ~1800 adults in England aged 16+. Thus, the same participants take part in both surveys. The respondents are recruited using a type of random location sampling, which is a hybrid between

For peer review only - http://bmjopen.bmj.com/site/about/guidelines.xhtml

random probability and simple quota sampling. England is first split into over 170000 'Output Areas', comprising of approximately 300 households. These areas are then stratified according to ACORN characteristics and geographic region (http://www.caci.co.uk/acorn/) and are randomly allocated to interviewers. Interviewers visit households within their allocated locality starting at a random point in the area. One member per household, chosen based on who the interviewer judge would best fulfil their quota requirements, is interviewed until interviewers achieve local quotas designed to minimise differences in the probability of participation. Participants appear to be representative of the population in England, having similar socio-demographic composition and smoking characteristics to large national surveys based on probability samples such as the Health Survey for England (23), while drinking characteristics also appear similar at a regional level to other national surveys (24). For further details see: www.smokinginengland.info and www.alcoholinengland.info and the published protocol (21, 23).

Participants

Data were collected on 88,122 participants over the study period. Of these, 19.94% (95%CI 19.67 to 20.20 n=17,560) reported that they had smoked in the past year. Forty-seven percent of past year smokers (n=8097) were male, 18.9% (n=3272) were aged 16-24, 19.7% (n=3416) were aged 25 to 34, 16.2% (n=2804) were aged 35 to 44, 17.0% (n=2946) were aged 45 to 54, 14.6% (n=2521) were aged 55 to 64 and 13.7% (n=2371) were aged 65+. Finally, 59.4% (n=10286) were in manual occupations. Data from these participants were aggregated monthly and this forms the basis of the sample in this paper.

Measures

Input series

Participants were asked whether they smoked or had smoked cigarettes (including hand-rolled) daily or non-daily in the past year and to complete the Alcohol Use Disorders Identification Test

For peer review only - http://bmjopen.bmj.com/site/about/guidelines.xhtml

(AUDIT) (25). The AUDIT identifies people who could be classed as dependent, harmful or hazardous drinkers and has demonstrated validity, high internal consistency and good test-retest reliability across gender, age and cultures (26-29). Those scoring 8 or more were classed as high-risk drinkers. This is a common threshold for high-risk drinkers (27, 30-32). The prevalence of high-risk alcohol consumption among smokers in each month was obtained by counting the number smokers reporting an AUDIT score greater than or equal to 8.

Output series

Past year smokers were then asked:

1. "How many serious attempts to stop smoking have you made in the last 12 months? By serious attempt I mean you decided that you would try to make sure you never smoked again. Please include any attempt that you are currently making and please include any successful attempt made within the last year".
2. "How long did your most recent serious quit attempt last before you went back to smoking?"

The prevalence of quit attempts in monthly were calculated as the number of respondents who reported having made one or more quit attempts in the past 12 months divided by the number of past year smokers. The success rate in each quarter was calculated as the number of respondents reporting that they were still not smoking divided by the number reporting having made a quit attempt.

Covariates

Past-year smokers' socio-economic status was assessed by social grade measured using the British National Readership Survey (NRS) Social Grade Classification Tool (27): AB (higher managerial, administrative or professional), C1 (supervisory or clerical and junior managerial, administrative or

For peer review only

For peer review only - http://bmjopen.bmj.com/site/about/guidelines.xhtml

professional), C2 (skilled manual workers), D (semi-skilled and unskilled manual workers) and E (casual or lowest grade workers, pensioners, and others who depend on the welfare state for their income). The prevalence of smokers in lower social grades in each quarter was calculated as the proportion of past-year smokers who reported being in C2, D and E. Past-year smokers were also asked their age, with a mean estimated each month.

Analysis

The analysis plan, data and syntax were preregistered on the Open Science Framework (https://osf.io/384gx/). An amendment was made to the analysis plan following reviewer comments to also adjust for socio-demographic variables. Variables can only be included in ARIMAX models at the aggregated level and must vary sufficiently over the study period (33)). There was insufficient variation in gender and ethnicity over the period but therewas sufficient variation in mean age and the proportion of those in lower social-grades, which were included. Studies have shown an increase in the age of smokers over time (34) and socio-economic status is a strong predictor of quitting activity (35, 36).

Cases with missing data on either smoking or drinking variables were classified as missing in calculating the prevalence figures: smoking status (n=55; %=0.06), high-risk drinking status among smokers (n=202; %=1.17) and quit attempts among smokers (n=562; %=3.24). All data were analysed in R studio.

Data were weighted (see (23) for further details) to match the population in England and analysed using Autoregressive Integrated Moving Average with Exogeneous Input (ARIMAX) modelling to assess the association between prevalence of high risk drinking among smokers and 1) prevalence of attempts to quit smoking and 2) prevalence of successful attempts to quit smoking among those having made a quit attempt. ARIMAX is an extension of autoregressive integrated moving average

For peer review only - http://bmjopen.bmj.com/site/about/guidelines.xhtml

analysis (ARIMA), which produces forecasts based upon prior values in the time series (Autoregressive terms; AR) and the errors made by previous predictions (Moving Average terms; MA). We followed a standard ARIMAX modelling approach (37).

The ARIMAX assumption of weak exogeneity was met: past prevalence of quit attempts (p=0.747) and quit success (p=0.999) did not statistically predict future prevalence of high-risk drinking among smokers. No outliers were identified in any of the series using an approach based on that described by Chen and Liu(38, 39). To stabilise the variance the series were log-transformed. The Augmented Dickey-Fuller test and visual inspection of the plots indicated that first order differencing was required for both time series. First order differencing involves calculating the change between one observation and the next. No additional seasonal differencing was required (40).

The autocorrelation and partial autocorrelation functions were examined to determine the non-seasonal MA and AR terms. These suggested an ARIMAX(0,1,1) model for the time series predicting both prevalence of quit attempts and prevalence of quit success. This was confirmed by comparing models with different specifications using the AIC. To identify the most appropriate transfer function for the continuous explanatory variables the sample cross-correlation function was checked and models with varying distributed lags compared using the Akaike Information Criterion. This suggested a lag of 0 when predicting the prevalence of quit attempts and predicting the prevalence of quit success, thus only current values and not lagged (past period) values of the input series were used to predict current values of the output series. In our previous study, prevalence of smoking was found to be associated with high-risk drinking with a distributed lag of 2 (22). Thus, additional sensitivity analyses were run with the output series lagged by an order of 2 i.e. the time base was shifted back by 2 months.

For peer review only - http://bmjopen.bmj.com/site/about/guidelines.xhtml

The Ljung-Box test for white noise showed that the residuals for both fitted models were free of serial correlation. A number of additional model checks were also made. First, the autocorrelation terms included in the model were checked for their statistical significance. Secondly, it was determined whether the model residuals were normally distributed, random and independent. Finally, that the inclusion of the MA term conformed to the bounds of invertibility i.e. its value was <1 (37, 38).

Bayes factors (BFs) were derived for non-significant findings using an online calculator to disentangle whether there is evidence for the null hypothesis of no effect (BF <1/3rd) or the data are insensitive (BF between 1/3rd and 3)(41, 42). A half-normal distribution was assumed with a percentage change in the outcomes of interest for every percentage increase in the input series of 0.6%. This is on the basis of a previous study showing that smokers who had made a quit attempt were around 40% less likely to report that they were high risk drinkers (9). Strengthening the Reporting of Observational Studies in Epidemiology (STROBE) guidelines for the reporting of observational studies were followed throughout (43).

Patient involvement

No patients were involved in setting the research question or the outcome measures, nor were they involved in developing plans for recruitment, design, or implementation of the study. No patients were asked to advise on interpretation or writing up of results. There are no plans to disseminate the results of the research directly to study participants or any specific patient community.

Results

Figure 1 shows the raw time series data from 2014 to 2018. Prevalence of high-risk drinking among smokers declined from 26.9% (95% CI 22.34 to 32.03) in 2014 to 23.7% (95% CI 19.26 to 28.87) in June 2018. Attempts to quit smoking also declined from 38.1% (95%CI 32.86 to 43.66) to 28.5%

For peer review only - http://bmjopen.bmj.com/site/about/guidelines.xhtml

(95%CI 23.60 to 33.90) and quit success from 19.6% (95%CI 13.22 to 27.87) to 9.4% (95%CI 4.51 to 17.95) in June 2018.

Tables 1 shows the results of the ARIMAX models assessing the association between prevalence of high-risk drinking among smokers and (1) quit attempts and (2) quit success. The findings were inconclusive as to whether any associations were present. BFs suggested that there is anecdotal evidence for the null hypothesis that prevalence of high-risk drinking among smokers is not associated with prevalence of quit attempts and prevalence of quit success. Findings were similar when a 2 month back shifted lag was used for prevalence of quit attempts and quit success. Adjusting for age and social-grade did not change the findings (see Table 2).

Discussion

To our knowledge, this is the first empirical study to estimate the population association between high-risk drinking among smokers and attempts to quit smoking and the success of those attempts. There was weak evidence that there was no substantial association between changes in the prevalence of high-risk drinking and quit attempts and quit success.

These findings appear to be at odds with individual level studies which suggest that smokers with an alcohol use disorder are less likely to attempt and succeed in stopping smoking (12, 13). Alcohol consumption during attempts at smoking cessation is also associated with a greater risk of relapse (14). As a result, smokers are often advised to lower their alcohol consumption when they attempt to quit smoking(9). Of course, it remains plausible that high-risk drinking among smokers may still be associated with a small effect on mean population prevalence of quit attempts and their success, but it was not possible to detect this in the current study. An association may also be masked by factors impacting at a population level which were not accounted for in the current study. Although we are unaware of any major population-level interventions or other events during the study period which may have affected the associations under investigation, we cannot rule out residual

For peer review only - http://bmjopen.bmj.com/site/about/guidelines.xhtml

confounding. There may also be some statistical bias due to the loss of power and sensitivity that comes with aggregating data. Prevalence of high-risk drinking among smokers will also be somewhat noisier than if prevalence was also assessed among non-smokers, given the smaller sample size involved in estimation.

These findings suggest that the previously identified positive association between prevalence of smoking and prevalence of high-risk drinking is unlikely to be causal, whereby smokers attempting to quit, and those succeeding, also reduce their alcohol intake (22). Although it remains possible that use of alcohol by smokers impacts on other key indices including longer term abstinence, the small proportion of smokers who relapse long term (i.e. after a year) could not account for the size of association noted. It may instead be that overall prevalence is related to an unmeasured variable, perhaps economic factors and sociocultural events, that account for the change in both smoking and high-risk drinking. For example, in recent years taxation on cigarettes and alcohol have increased linearly, driving down sales of both (44, 45). There have also been substantial fluctuations in average household income since 2013, which have been shown to independently affect smoking and alcohol consumption (46-48). Sporting events such as the Olympics may also concurrently increase alcohol and tobacco intake as they are celebratory occasions. Mass media campaigns may also play role, simultaneously promoting attempts to quit smoking and the adoption of a healthier lifestyle by reducing alcohol intake (49).

For peer review only

A strength of this study is the use of a large representative sample of the population in England. Several limitations need to be considered. First, the ATS required participants to recall their alcohol consumption and attempts to quit smoking which is likely to have been somewhat inaccurate due to recall bias and social desirability. For example, it has been found that a large proportion of unsuccessful quit attempts fail to be reported, particularly if they only last a short time or occurred longer ago(50). However, social pressure in population surveys tends to be low and so it is generally considered acceptable to rely on self-report data(51). Second, these findings may not

For peer review only - http://bmjopen.bmj.com/site/about/guidelines.xhtml

generalise to other countries. England has a strong tobacco-control climate. In countries with weaker tobacco control or different alcohol control policies, different effects may be observed. Thirdly, this paper did not consider the impact of changes in excessive alcohol consumption prevalence on the length of quit success, being defined as having made a quit in attempt in the last 12 months and still reporting not smoking. This will be an important area for future research as more data are accumulated to provide adequate power. Finally, although there can be no individual-level confounding in population trend data there is a possibility of population-level confounding, such as introduction of policies that may affect quitting rates. However, we were unable to identify any such population policies occurring during the study period that may have confounded the results.

Conclusion

These findings suggest that the previously identified positive association between prevalence of smoking and prevalence of high-risk drinking is unlikely to be causal, whereby smokers attempting to quit, and those succeeding, also reduce their alcohol intake. Instead, it may be that overall prevalence is related to an unmeasured third variable such as economic factors and sociocultural events.

For peer review only - http://bmjopen.bmj.com/site/about/guidelines.xhtml

List of abbreviations

ARIMAX - autoregressive integrated moving average with exogenous input
BF – Bayes Factor
ATS – Alcohol Toolkit Study
STS – Smoking Toolkit Study

For peer review only

For peer review only - http://bmjopen.bmj.com/site/about/guidelines.xhtml

Table 1: Estimated percentage point changes in proportion of quit attempts and proportion of quitters who met criteria for quit success during the study period, based on autoregressive integrated moving average with exogenous input (ARIMAX) models

		Output series					
		Quit attempts[1]			Quit success[2]		
		Percentage change per 1% change in the exposure	95%CI	p	Percentage change per 1% change in the exposure	95%CI	p
Input series	**Model 1:** High-risk drinking among smokers (no backward lag of the output series)	0.156	-0.079 to 0.391	0.194	0.066	-0.524 to 0.655	0.827
	**Model 2:** High-risk drinking among smokers (two month backward lag of the output series)	0.065	-0.183 to 0.313	0.608	0.134	-0.469 to 0.736	0.663
	**Bayes Factor**						
	Model 1	0.80			0.53		
	Model 2	0.33			0.64		

Table 2: Estimated percentage point changes in proportion of quit attempts and proportion of quitters who met criteria for quit success during the study period, based on autoregressive integrated moving average with exogenous input (ARIMAX) models – adjusted age and social-economic status

		Output series					
		Quit attempts[1]			Quit success[2]		
		Percentage change per 1% change in the exposure	95%CI	p	Percentage change per 1% change in the exposure	95%CI	p
Input series	**Model 1:** High-risk drinking among smokers (no backward lag of the output series)	0.040	-0.214 to 0.294	0.758	0.168	-0.489 to 0.825	0.616
	**Model 2:** High-risk drinking among smokers (two month backward lag of the output series)	0.030	-0.229 to 0.289	0.822	0.132	-0.549 to 0.814	0.703

Figure 1: Prevalence of a) high-risk drinking; b) attempts to quit smoking and c) quit success

Declarations

Ethical approval and consent to participate

For peer review only - http://bmjopen.bmj.com/site/about/guidelines.xhtml

For peer review only

Ethics approval for the Smoking Toolkit Survey (STS) was originally granted by the UCL Ethics Committee (ID 0498/001) and approval for the ATS was granted by the same committee as an extension of the STS (ID 2808/005). In accordance with our ethical approval, all respondents were given a written information sheet about the study, and provided informed verbal consent

Availability of data and material

The analysis plan, data and syntax were preregistered on the Open Science Framework (https://osf.io/384gx/).

Funding

The Smoking Toolkit Study is currently primarily funded by Cancer Research UK (C1417/A14135; C36048/A11654; C44576/A19501) and has previously also been funded by Pfizer, GSK, and the Department of Health. The ATS is currently funded by the NIHR School for Public Health Research (SPHR) (SPHR-SWP-ALC-WP5). SPHR is a partnership between the Universities of Sheffield; Bristol; Cambridge; Exeter; UCL; The London School for Hygiene and Tropical Medicine; the LiLaC collaboration between the Universities of Liverpool and Lancaster and Fuse; The Centre for Translational Research in Public Health, a collaboration between Newcastle, Durham, Northumbria, Sunderland and Teesside Universities. The views expressed are those of the authors(s) and not necessarily those of the NHS, NIHR, or Department of Health. No funders had any involvement in the design of the study, the analysis or interpretation of the data, the writing of the report, or the decision to submit the paper for publication. JB's post is funded CRUK (C1417/A14135). RW is funded by Cancer Research UK (C1417/A14135). EB is funded by the NIHR SPHR (SPHR-SWP-ALC-WP5) and CRUK also provide support (C1417/A14135).

Competing interests

RW undertakes consultancy and research for and receives travel funds and hospitality from manufacturers of smoking cessation medications. RW salary is funded by Cancer Research UK. SM receives support from Cancer Research UK and the National Institute for Health Research (NIHR)'s School for Public Health Research (SPHR). EB and JB have received unrestricted research funding from Pfizer. PM's research is funded by a variety of governmental funding agencies including UKRI and NIHR.

Authors' contributions

EB, JB, SM and RW wrote the first draft of the manuscript and conducted the analysis. All other authors commented on this draft and contributed to the final version. All authors read and approved the final manuscript.

For peer review only - http://bmjopen.bmj.com/site/about/guidelines.xhtml

References

1. Statistics OfN. Adult smoking habits in the UK: 2017. 2018.
2. Health and Social Care Information Centre. Statistics on Alcohol, England 2016 2016 [Available from: https://www.gov.uk/government/statistics/statistics-on-alcohol-england-2016.
3. Kvaavik E, Batty GD, Ursin G, Huxley R, Gale CR. Influence of individual and combined health behaviors on total and cause-specific mortality in men and women: the United Kingdom health and lifestyle survey. Archives of internal medicine. 2010;170(8):711-8.
4. Lasser K, Boyd JW, Woolhandler S, Himmelstein DU, McCormick D, Bor DH. Smoking and mental illness: a population-based prevalence study. JAMA. 2000;284.
5. Falk DE, Yi HY, Hiller-Sturmhofel S. An epidemiologic analysis of co-occurring alcohol and tobacco use and disorders: findings from the National Epidemiologic Survey on Alcohol and Related Conditions. Alcohol Res Health. 2006;29.
6. Kalman D, Morissette SB, George TP. Co-morbidity of smoking in patients with psychiatric and substance use disorders. Am J Addict. 2005;14.
7. Kessler RC, Chiu WT, Demler O, Merikangas KR, Walters EE. Prevalence, severity, and comorbidity of 12-month DSM-IV disorders in the National Comorbidity Survey Replication. Arch Gen Psychiatry. 2005;62.
8. Hasin DS, Stinson FS, Ogburn E, Grant BF. Prevalence, correlates, disability, and comorbidity of DSM-IV alcohol abuse and dependence in the United States: results from the National Epidemiologic Survey on Alcohol and Related Conditions. Arch Gen Psychiatry. 2007;64.
9. Brown J, West R, Beard E, Brennan A, Drummond C, Gillespie D, et al. Are recent attempts to quit smoking associated with reduced drinking in England? A cross-sectional population survey. BMC Public Health. 2016;16(1):1-7.
10. Przulj D, Hajek P, Snuggs S, McRobbie H. Changes in Alcohol Consumption During a Stop-Smoking Attempt and Differences Between Smokers Using Nicotine Replacement and Smokers Using Varenicline. Nicotine Tob Res. 2018;20(5):583-8.
11. Hughes JR, Kalman D. Do smokers with alcohol problems have more difficulty quitting? Drug Alcohol Depend. 2006;82.
12. Weinberger AH, Pilver CE, Hoff RA, Mazure CM, McKee SA. Changes in smoking for adults with and without alcohol and drug use disorders: longitudinal evaluation in the U.S. population. Am J Drug Alcohol Abuse. 2013;39.
13. Leeman RF, McKee SA, Toll BA, Krishnan-Sarin S, Cooney JL, Makuch RW, et al. Risk factors for treatment failure in smokers: relationship to alcohol use and to lifetime history of an alcohol use disorder. Nicotine Tob Res. 2008;10.
14. Kahler CW, Spillane NS, Metrik J. Alcohol use and initial smoking lapses among heavy drinkers in smoking cessation treatment. Nicotine Tob Res. 2010;12.
15. Gulliver SB, Kamholz BW, Helstrom AW. Smoking cessation and alcohol abstinence: what do the data tell us? Alcohol Res Health. 2006;29.
16. Friend KB, Pagano ME. Smoking cessation and alcohol consumption in individuals in treatment for alcohol use disorders. J Addict Dis. 2005;24.
17. Rose JE, Brauer LH, Behm FM, Cramblett M, Calkins K, Lawhon D. Psychopharmacological interactions between nicotine and ethanol. Nicotine & Tobacco Research. 2004;6(1):133-44.
18. Field M, Mogg K, Bradley BP. Alcohol increases cognitive biases for smoking cues in smokers. Psychopharmacology. 2005;180(1):63-72.
19. Verplaetse TL, McKee SA. An overview of alcohol and tobacco/nicotine interactions in the human laboratory. The American journal of drug and alcohol abuse. 2017;43(2):186-96.
20. Blume AW, Schmaling KB, Marlatt GA. Revisiting the self-medication hypothesis from a behavioral perspective. Cognitive and Behavioral Practice. 2000;7(4):379-84.
21. Beard E, Brown J, West R, Acton C, Brennan A, Drummond C, et al. Protocol for a national monthly survey of alcohol use in England with 6-month follow-up:'The Alcohol Toolkit Study'. BMC public health. 2015;15(1):230.

For peer review only - http://bmjopen.bmj.com/site/about/guidelines.xhtml

22. Beard E, West R, Michie S, Brown J. Association between smoking and alcohol-related behaviours: a time–series analysis of population trends in England. Addiction (Abingdon, England). 2017;112(10):1832-41.
23. Fidler JA, Shahab L, West O, Jarvis MJ, McEwen A, Stapleton JA, et al. 'The smoking toolkit study': a national study of smoking and smoking cessation in England. BMC Public Health. 2011;11(1):479.
24. Beard E, Brown J, West R, Angus C, Kaner E, Michie S. Healthier central England or North–South divide? Analysis of national survey data on smoking and high-risk drinking. BMJ Open. 2017;7(3).
25. Casswell S, Meier P, MacKintosh AM, Brown A, Hastings G, Thamarangsi T, et al. The International Alcohol Control (IAC) Study—Evaluating the Impact of Alcohol Policies. Alcoholism: Clinical and Experimental Research. 2012;36(8):1462-7.
26. Saunders JB, Aasland OG, Amundsen A, Grant M. Alcohol consumption and related problems among primary health care patients: WHO collaborative project on early detection of persons with harmful alcohol consumption--I. Addiction (Abingdon, England). 1993;88(3):349-62.
27. Saunders JB, Aasland OG, Babor TF, de la Fuente JR, Grant M. Development of the Alcohol Use Disorders Identification Test (AUDIT): WHO Collaborative Project on Early Detection of Persons with Harmful Alcohol Consumption--II. Addiction (Abingdon, England). 1993;88(6):791-804.
28. Allen JP, Litten RZ, Fertig JB, Babor T. A Review of Research on the Alcohol Use Disorders Identification Test (AUDIT). Alcoholism: Clinical and Experimental Research. 1997;21(4):613-9.
29. Hays R, Merz J, Nicholas R. Response burden, reliability, and validity of the CAGE, Short MAST, and AUDIT alcohol screening measures. Behavior Research Methods, Instruments, & Computers. 1995;27(2):277-80.
30. Caviness CM, Hatgis C, Anderson BJ, Rosengard C, Kiene SM, Friedmann PD, et al. Three Brief Alcohol Screens for Detecting Hazardous Drinking in Incarcerated Women. Journal of Studies on Alcohol and Drugs. 2009;70(1):50-4.
31. National Institute on Alcohol Abuse and Alcoholism. Helping Patients Who Drink Too Much: A Clinician's Guide. Bethesda, MD: National Institute on Alcohol Abuse and Alcoholism; 2007.
32. Kokotailo PK, Egan J, Gangnon R, Brown D, Mundt M, Fleming M. Validity of the alcohol use disorders identification test in college students. Alcoholism: Clinical and Experimental Research. 2004;28(6):914-20.
33. Beard E, Marsden J, Brown J, Tombor I, Stapleton J, Michie S, et al. Understanding and using time series analyses in addiction research. Addiction. 2019;114(10):1866-84.
34. Garnett C, Tombor, I., Beard, E., Jackson, S. E., West, R., & Brown, J. Have smoking characteristics and quitting behaviour changed between 2008 and 2017 in England? A population study. Submitted.
35. Hiscock R, Judge K, Bauld L. Social inequalities in quitting smoking: what factors mediate the relationship between socioeconomic position and smoking cessation? Journal of Public Health. 2011;33(1):39-47.
36. Parks MJ, Kingsbury JH, Boyle RG, Choi K. Behavioral change in response to a statewide tobacco tax increase and differences across socioeconomic status. Addict Behav. 2017;73:209-15.
37. Box GE, Jenkins GM, Reinsel GC. Time series analysis: forecasting and control: John Wiley & Sons; 2011.
38. Chen C, Liu L-M. Joint estimation of model parameters and outlier effects in time series. Journal of the American Statistical Association. 1993;88(421):284-97.
39. López-de-Lacalle J. tsoutliers R Package for Automatic Detection of Outliers in Time Series.
40. Dickey DA, Fuller WA. Distribution of the estimators for autoregressive time series with a unit root. Journal of the American statistical association. 1979;74(366a):427-31.

For peer review only - http://bmjopen.bmj.com/site/about/guidelines.xhtml

41. Dienes Z. Using Bayes to get the most out of non-significant results. Frontiers in Psychology. 2014;5(781).
42. Beard E, Dienes Z, Muirhead C, West R. Using Bayes factors for testing hypotheses about intervention effectiveness in addictions research. Addiction (Abingdon, England). 2016;111(12):2230-47.
43. Von Elm E, Altman DG, Egger M, Pocock SJ, Gøtzsche PC, Vandenbroucke JP, et al. The Strengthening the Reporting of Observational Studies in Epidemiology (STROBE) statement: guidelines for reporting observational studies. PLoS medicine. 2007;4(10):e296.
44. Institute of Alcohol Studies. How have UK alcohol taxes changed over time? 2017 [Available from: http://www.ias.org.uk/Alcohol-knowledge-centre/Price/Factsheets/How-have-UK-alcohol-taxes-changed-over-time.aspx.
45. ASH. Timeline of changes in tobacco taxation in the UK from 1993 to the present 2017 [Available from: http://ash.org.uk/information-and-resources/taxation-illicit-trade/taxation/ash-analysis-of-tobacco-tax-increases-in-the-united-kingdom/.
46. Cerdá M, Johnson-Lawrence V, Galea S. Lifetime income patterns and alcohol consumption: Investigating the association between long- and short-term income trajectories and drinking. Social science & medicine (1982). 2011;73(8):1178-85.
47. Perelman J, Alves J, Pfoertner TK, Moor I, Federico B, Kuipers MAG, et al. The association between personal income and smoking among adolescents: a study in six European cities. Addiction (Abingdon, England). 2017;112(12):2248-56.
48. ONS. Nowcasting household income in the UK: financial year ending 2017. 2018.
49. Wakefield MA, Loken B, Hornik RC. Use of mass media campaigns to change health behaviour. The Lancet. 2010;376(9748):1261-71.
50. Berg CJ, An LC, Kirch M, Guo H, Thomas JL, Patten CA, et al. Failure to report attempts to quit smoking. Addictive behaviors. 2010;35(10):900-4.
51. Wong SL, Shields M, Leatherdale S, Malaison E, Hammond D. Assessment of validity of self-reported smoking status. Health reports. 2012;23(1):D1.

For peer review only

For peer review only - http://bmjopen.bmj.com/site/about/guidelines.xhtml

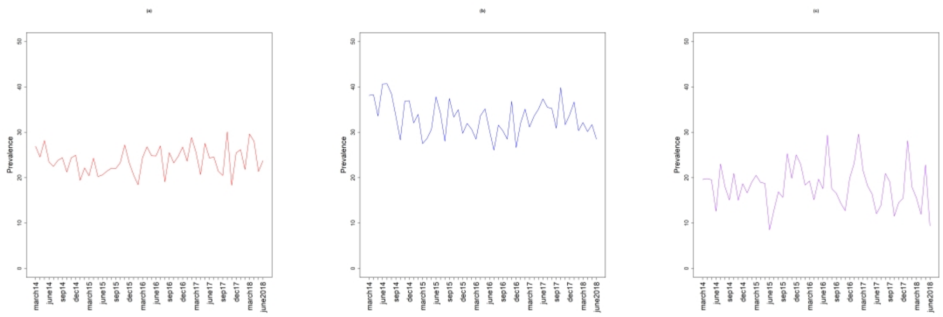

762x254mm (300 x 300 DPI)

STROBE 2007 (v4) Statement—Checklist of items that should be included in reports of *cross-sectional studies*

Section/Topic	Item #	Recommendation	Reported on page #
**Title and abstract**	1	(*a*) Indicate the study's design with a commonly used term in the title or the abstract	1
		(*b*) Provide in the abstract an informative and balanced summary of what was done and what was found	2
**Introduction**			
Background/rationale	2	Explain the scientific background and rationale for the investigation being reported	4-5
Objectives	3	State specific objectives, including any prespecified hypotheses	5
**Methods**			
Study design	4	Present key elements of study design early in the paper	6
Setting	5	Describe the setting, locations, and relevant dates, including periods of recruitment, exposure, follow-up, and data collection	6
Participants	6	(*a*) Give the eligibility criteria, and the sources and methods of selection of participants	6
Variables	7	Clearly define all outcomes, exposures, predictors, potential confounders, and effect modifiers. Give diagnostic criteria, if applicable	6-7
Data sources/ measurement	8*	For each variable of interest, give sources of data and details of methods of assessment (measurement). Describe comparability of assessment methods if there is more than one group	6-7
Bias	9	Describe any efforts to address potential sources of bias	*7*
Study size	10	Explain how the study size was arrived at	6-7
Quantitative variables	11	Explain how quantitative variables were handled in the analyses. If applicable, describe which groupings were chosen and why	6-7
Statistical methods	12	(*a*) Describe all statistical methods, including those used to control for confounding	6-7
		(*b*) Describe any methods used to examine subgroups and interactions	6-8
		(*c*) Explain how missing data were addressed	6-8
		(*d*) If applicable, describe analytical methods taking account of sampling strategy	6-8
		(*e*) Describe any sensitivity analyses	6-8
**Results**			

For peer review only

Participants	13*	(a) Report numbers of individuals at each stage of study—eg numbers potentially eligible, examined for eligibility, confirmed eligible, included in the study, completing follow-up, and analysed	9
		(b) Give reasons for non-participation at each stage	N/a
		(c) Consider use of a flow diagram	N/a
Descriptive data	14*	(a) Give characteristics of study participants (eg demographic, clinical, social) and information on exposures and potential confounders	
		(b) Indicate number of participants with missing data for each variable of interest	N/a
Outcome data	15*	Report numbers of outcome events or summary measures	9-10
Main results	16	(*a*) Give unadjusted estimates and, if applicable, confounder-adjusted estimates and their precision (eg, 95% confidence interval). Make clear which confounders were adjusted for and why they were included	9-10
		(*b*) Report category boundaries when continuous variables were categorized	9-10
		(*c*) If relevant, consider translating estimates of relative risk into absolute risk for a meaningful time period	NA
Other analyses	17	Report other analyses done—eg analyses of subgroups and interactions, and sensitivity analyses	9-10
**Discussion**			
Key results	18	Summarise key results with reference to study objectives	10-11
Limitations	19	Discuss limitations of the study, taking into account sources of potential bias or imprecision. Discuss both direction and magnitude of any potential bias	10-11
Interpretation	20	Give a cautious overall interpretation of results considering objectives, limitations, multiplicity of analyses, results from similar studies, and other relevant evidence	10-11
Generalisability	21	Discuss the generalisability (external validity) of the study results	10-11
**Other information**			
Funding	22	Give the source of funding and the role of the funders for the present study and, if applicable, for the original study on which the present article is based	14

*Give information separately for cases and controls in case-control studies and, if applicable, for exposed and unexposed groups in cohort and cross-sectional studies.

Note: An Explanation and Elaboration article discusses each checklist item and gives methodological background and published examples of transparent reporting. The STROBE checklist is best used in conjunction with this article (freely available on the Web sites of PLoS Medicine at http://www.plosmedicine.org/, Annals of Internal Medicine at http://www.annals.org/, and Epidemiology at http://www.epidem.com/). Information on the STROBE Initiative is available at www.strobe-statement.org.

For peer review only
